# Differences in schizophrenia treatments by race and ethnicity—analysis of electronic health records

**DOI:** 10.1038/s41537-024-00470-4

**Published:** 2024-04-26

**Authors:** Candice Medina, Ayooluwa Akinkunmi, Nevaeh Bland, Eva Velthorst, Avi Reichenberg, Vahe Khachadourian, Amirhossein Modabbernia, Magdalena Janecka

**Affiliations:** 1 Department of Psychiatry, Icahn School of Medicine at Mount Sinai, New York, NY USA; 2 Seaver Autism Center for Research and Treatment, Icahn School of Medicine at Mount Sinai, New York, NY USA; 3 Department of Child and Adolescent Psychiatry, NYU Grossman School of Medicine, New York, NY USA; 4 Haverford College, Haverford, PA USA; 5 GGZ Noord Holland Noord, Stationsplein, Heerhugowaard Netherlands; 6 Department of Environmental Medicine, Icahn School of Medicine at Mount Sinai, New York, NY USA; 7 Department of Genetic & Genomic Sciences, Icahn School of Medicine at Mount Sinai, New York, NY USA; 8 Department of Population Health, NYU Grossman School of Medicine, New York, NY USA

**Keywords:** Schizophrenia, Psychosis

## Abstract

Racial disparities in prescriptions of anti-psychotics have been highlighted before. However, (i) the evidence on other medications, including anti-depressant or mood stabilizing medications is lacking, and (ii) the role of potentially confounding factors and (iii) specificity of such disparities to schizophrenia (SCZ), are still unknown. We used electronic health records (EHRs) from 224,212 adults to estimate the odds ratios of receiving a prescription for different nervous system medications among patients with SCZ of different race/ethnicity, and analogous linear models to investigate differences in prescribed medication doses. To verify specificity of the observed patterns to SCZ, we conducted analogous analyses in depression and bipolar disorder (BD) patients. We found that Black/African American (AA) and Hispanic patients with SCZ were more likely to be prescribed haloperidol (Black/AA: OR = 1.52 (1.33–1.74); Hispanic: OR = 1.32 (1.12–1.55)) or risperidone (Black/AA: OR = 1.27 (1.11–1.45); Hispanic: OR = 1.40 (1.19–1.64)), but less likely to be prescribed clozapine (Black/AA: OR = 0.40 (0.33-0.49); Hispanic: OR = 0.45 (0.35-0.58)), compared to white patients. There were no race/ethnicity-related differences in the prescribed medication doses. These patterns were not specific to SCZ: Asian, Hispanic and Black/AA patients with BD or depression were more likely to be prescribed anti-psychotics, but less likely to be prescribed antidepressants or mood-stabilizers. In conclusion, we found racial/ethnic disparities in the medications prescribed to patients with SCZ and other psychiatric conditions. We discuss the potential implications for the quality of care for patients of diverse races/ethnicities.

## Introduction

Studies have reported significant racial and ethnic disparities in the diagnosis and treatment of numerous mental health conditions. Patients from racial/ethnic minorities are 3-4 times more likely to be diagnosed with schizophrenia^1–6^, with similar disparities reported across a broad range of mental health conditions in both adult^7^, and pediatric populations^8^.

The treatment of psychotic disorders also varies by race and ethnicity. Black/African American (AA) and Hispanic individuals are less likely to have access to mental health services^9,10^, or to receive adjunctive psychopharmacological treatment^11,12^, but tend to receive higher doses of antipsychotics compared to white patients^13^. Taken together, evidence suggests that Black/AA and Hispanic patients with psychotic disorders receive different medications, receive them at different doses, and are less likely to be offered non-pharmacological treatments such as cognitive behavioral or family therapy^14^.

These discrepancies are critical to address: suboptimal treatment can hinder symptom management, leading to adverse side effects and medication non-compliance^15–17^, with wide-ranging implications for quality of life. Understanding how treatments differ among patients of diverse race/ethnicities, the origins of these disparities, and their consequences for clinical prognosis, is urgently needed for providing equitable healthcare, and advancing more broadly defined social justice (e.g., through improving employment opportunities through adequate symptom management). Prior studies investigating such differences were limited in their sample size and lacked data on potential effect modifiers and confounders, e.g., insurance status. Furthermore, they often lacked broader prescription and diagnostic data, precluding conclusions about adjunctive medications, and adjustment for comorbid psychiatric diagnoses.

To overcome these limitations, we utilized comprehensive data on clinical, demographic and administrative factors available in the EHRs of one of the largest and most diverse hospital systems in New York. This allowed us to advance prior findings by (1) testing the differences in the likelihood of being prescribed certain medications, and their dose, by patients’ race/ethnicity, accounting for diverse clinical, demographic and administrative factors, and (2) interrogate the extent to which these effects generalize across different psychiatric disorders. While earlier studies tended to focus on racial disparities in the context of specific mental health diagnosis (e.g. schizophrenia), we wanted to investigate whether these observations are pertinent to a single diagnosis, or reflect broader perceptions of race and ethnicity in mental health care. Using data from the same health network, and applying standardized analytical procedures, we therefore compared the prescription patterns among patients with schizophrenia, depression and bipolar disorder.

## Methods

### Data sources

Demographic, clinical, medication and administrative data on all patients with an ICD-10 diagnosis of SCZ, BD or depression by a Mount Sinai Health System (MSHS) provider. Data was extracted from the Epic EHRs by Mount Sinai Data Warehouse and fully de-identified at the point of the analysis. The study was reviewed and determined as human research exempt by the Program for the Protection of Human Subjects, Institutional Review Board for MSHS.

### Patient data

Patient data included: sex and self-reported race/ethnicity (Hispanic; Black/AA; White, Asian; American Indian or Alaska Native; Native Hawaiian or Pacific Islander; Other; Unknown; patients could indicate only one category); age (for de-identification, all patients aged >89 had their age set to 90), height, weight, insurance information (categorized as: self-pay; private insurance; Medicare; Medicaid; other federal coverage; unknown), ICD-10 codes related to mental health diagnosis (schizophrenia (F20; yes/no), bipolar disorder (F31; yes/no) and/or depression (F32, F33; yes/no)). NB. When a patient experiencing psychosis also presents with depressive episodes, manic episodes, or both, some clinicians may diagnose comorbid schizophrenia and bipolar disorder, while others may diagnose these symptoms under the umbrella of schizoaffective disorder. Importantly, schizophrenia and bipolar disorder are often not temporally co-occurring diagnosis. Whether an individual receives a dual diagnosis of schizophrenia and bipolar or a diagnosis of schizoaffective disorder depends on the evaluating clinician and the timing of the diagnosis. It is not uncommon for a patient to receive different diagnosis across multiple admissions, and some diagnoses may be based on medical records from other sources. In the absence of more detailed clinical notes, we decided against labeling these individuals as “schizoaffective” and chose instead to analyze their data as separate diagnostic categories of schizophrenia and bipolar disorder.

### Medication data

We recorded all prescriptions of qualifying nervous system medications in in-/outpatient, and emergency settings, including: mood stabilizers (ATC: N03A), antipsychotics (ATC: N05A), and antidepressants (ATC: N06A). We obtained information on the medication name and ATC code, prescribed maximum dose (mg or mg/ml) and frequency (times/day), route of administration, extended-release (yes/no), *pro re nata* (PRN) status (yes/no), and location of the provider.

All medications were grouped by their 7-digit ATC code, allowing us to analyze brand-name and generic forms of the same formulation together (e.g., Prozac and Sarafem (both ATC: N06AB03)). To calculate the total maximum daily dose for each patient, we multiplied patient’s highest recorded prescribed dose by the daily frequency. Due to uncertainty in the administered dose of the PRN medications, we excluded those from the analyses.

### Data quality control

We excluded all records of prescriptions with use frequency of >12/day. Due to lapses in weight unit recording, we only considered height and weight if BMI calculated assuming either imperial or metric system fell into 17-50 range, and weight into 40–204 (kg) or 90–450 (lbs) range. To reduce the ascertainment of patients receiving their pharmacotherapy outside of the MSHS, we excluded all individuals with a qualifying diagnosis but with no prescriptions of qualifying medications at MSHS.

### Statistical analyses

We conducted two sets of analyses: (1) addressing the question whether patients with schizophrenia of certain race/ethnicity are more/less likely to receive a prescription of some medications; and (2) addressing the question of whether, once prescribed the medication, patients of certain race/ethnicity tend to receive it at a higher/lower dose.

#### Medication rates

To compare medication rates across patients with different self-reported ethnicity/race, we used logistic mixed model regression, including a random effect of the provider location around the intercept (*lme4* R package^18^). We fitted a separate model for each unique medication, with prescription for that medication (yes/no) as an outcome and patient’s self-reported race/ethnicity as the key predictor. To ensure reliable coefficient estimation, we analyzed only the medications prescribed to at least 10 patients of certain ethnicity/race – therefore, as sample size differed across those groups, the number of medications analyzed differed between race/ethnicity groups. All analyses were adjusted for patient sex, age, known psychiatric comorbidities (depression, bipolar disorder) and insurance status, and accounted for the provider’s location by including it as a random effect around the intercept. We accounted for multiple testing using the False Discovery Rate^19^, setting the significance threshold for the q-value at 0.05.

##### Medication rates: additional analyses

First, we explored if weight and/or BMI could influence the pattern of prescriptions in our sample. While correlated, patient’s weight and BMI could independently influence both the medications they receive and their doses. Given high missingness of height and weight information (~16% sample), this was performed as an additional sensitivity analysis to avoid substantial sample loss in the primary analyses.

Secondly, to investigate if potential differences in prescription rates are due to different pattern of comorbidities by race/ethnicity, we repeated the main analyses in the subsample of patients without recorded depression or BD (NB. as SCZ and BD cannot be diagnosed concurrently, they are not comorbidities; however, as some patients with SCZ are initially misdiagnosed with BD^20^, this analysis allowed us to ensure that our results are robust to excluding patients with potentially more ambiguous symptoms).

Next, we tested if the differences in the rates of prescription of specific medications (7-digit ATC code) map onto differences in prescribing medications from broader therapeutic groups (4-digit ATC code, e.g. N06A for anti-depressants).

Finally, to investigate if potential differences in prescription rates are specific to SCZ, or generalize to other mental health outcomes, we conducted two additional sets of analyses for BD and depression. These analyses were analogous to the main (SCZ) analysis, but in each we restricted the sample to patients diagnosed with the respective disorder, using the remaining two diagnoses as covariates in the regression models.

#### Medication dose

The dose analyses were analogous to those for medication rates, using linear mixed model for continuous outcomes (daily medication dose). To calculate the total maximum daily dose for each patient, we multiplied the highest dose we have recorded for that patient in our dataset, by the daily frequency, and standardized it by converting it to a Z-score. We did not account for how long this dose was maintained. Only patients receiving the given medication could be included in the dose analyses, therefore the analytical sample size differed between medications, and we only conducted the analyses for the medications prescribed to at least 10 patients of given race/ethnicity. The key predictor was the self-reported race/ethnicity, and the covariates included sex, age, known psychiatric comorbidities, insurance status and whether the medication was prescribed in an extended release form (yes/no), accounting for the fact that certain medications in our dataset can be prescribed e.g. in both oral and long acting injectable (LAI) form. Additional analyses were run analogously to those described for the medication rates and included: (1) including weight and BMI as covariates; (2) excluding patients with comorbid depression/BD; (3) conducting analogous analyses for patients with depression and BD.

## Results

We obtained data on 224,212 patients diagnosed with SCZ, BD or depression within MSHS. Of those, we excluded 2 patients declining to provide race/ethnicity information and 2 providing different information on different occasions; 144,970 patients had data available on at least one qualifying prescription, and these constituted our analytical sample. White patients constituted the largest subgroup (*N* = 65,811; 45%), followed by Hispanic (*N* = 19,442; 13%), Black/AA (*N* = 17,669; 12%) and patients reporting ‘Other’ race/ethnicity (*N* = 14,774). Patients with no information on race/ethnicity (*N* = 23,508) were included in the analyses as ‘Unknown’ race/ethnicity.

Demographic information on patients with SCZ (*N* = 9104), who constituted the primary analysis group, is presented in Table 1. Among those patients, 36% indicated their race/ethnicity as Black/AA (*N* = 3264 (36%)). The sample of American Indian or Alaska Native, and Native Hawaiian or Pacific Islander patients with SCZ (>10 each) was too small for the analysis of medication patterns, and therefore individuals in these race/ethnicity categories were excluded from further analyses and their data is not presented for privacy protection. Demographic information on patients with depression (*N* = 125,743) and BD (*N* = 18,755) is available in Tables S1, S2.

**Table 1:** Characteristics of patients with SCZ diagnosis (ICD-10: F20).

	White [ref]	Black / African American	Hispanic	Asian	Other	Unknown
N	1682	3264	1340	252	1446	1110
% Female	40.2	40.4	43.4	48.4	38.8	37.8
Mean age (SD)	59.8 (16.8)	51.7 (16.4)	52.4 (17.2)	48.5 (16.4)	51.2 (17.2)	55.8 (16.9)
% depression	27.1	22.8	36.8	23.8	26.7	17.8
% bipolar disorder	15.0	16.1	19.4	9.1	15.5	10.2
% private insurance	3.0	5.0	5.3	5.6	5.6	3.2
% prescribed a mood stabilizer (N03A)	10.7	9.2	10.4	9.1	10.0	6.6
% prescribed an anti-psychotic (N05A)	91.3	93.0	90.7	94.0	91.6	90.1
% prescribed an anti-depressant (N06A)	49.0	38.1	55.2	40.5	43.5	38.0
Mean BMI (SD)	26.5 (6.7)	26.7 (6.8)	26.6 (6.8)	26.7 (7.0)	26.4 (6.8)	26.8 (6.7)

White patients serve as the reference (ref) group. Mean calculations are followed by standard deviations (SD).

### Medication rates

**Fig. 1:**
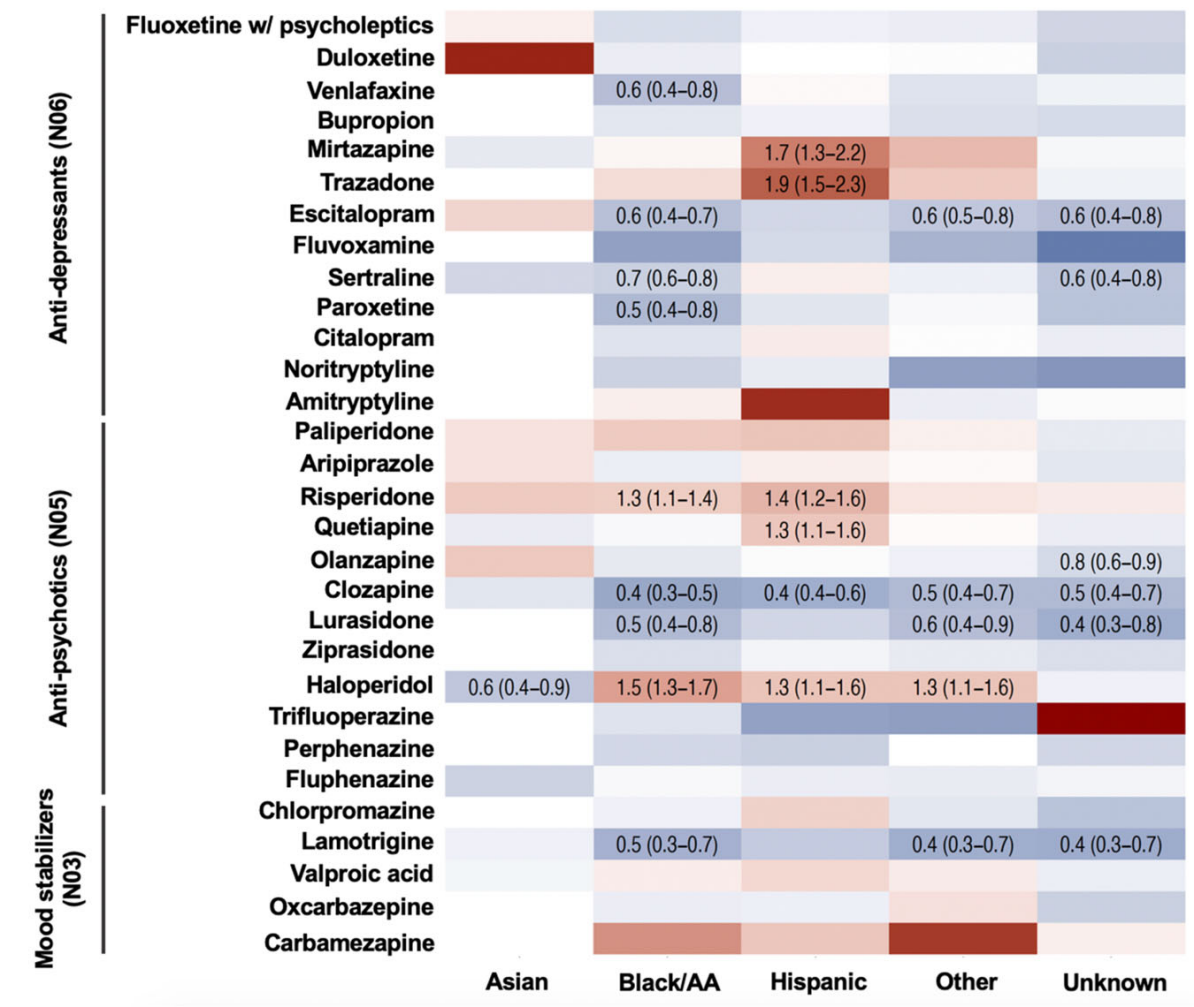
Likelihood of receiving specific anti-psychotic, anti-depressant and mood stabilizing medications in Asian, Black/AA, Hispanic, Other race/ethnicity and unknown race/ethnicity patients with SCZ diagnosis, compared to white patients. Red tiles indicate higher, and blue tiles lower likelihood of receiving the prescription of the given medication. Odds ratios and their 95% confidence intervals are presented only for the associations significant after multiple testing correction (false discovery rate). All analyses are adjusted for patient sex, age, comorbid depression and BD, and account for the location of the provider. Full results, including the non-significant findings, confidence intervals and number of patients with each prescription are presented in Supplemental Table S3.

Of the 48 qualifying medications recorded in patients with SCZ at MSHS, 16 medications had sufficient frequencies for analyses in Asian, and 30 in in the remaining race/ethnicity groups of patients. For these medications, we observed multiple differences in the prescription rates across race/ethnicity categories (Fig. 1; Table S3). Many of these differences were consistent between Hispanic and Black/AA patients, including: increased likelihood of being prescribed haloperidol (Black/AA: OR = 1.52(1.33–1.74), *P* = 1.82*10^–9^; Hispanic: OR = 1.32(1.12–1.55), *P* = 9.66*10^–4^) or risperidone (Black/AA: OR = 1.27(1.11–1.45), *P* = 5.37*10^–4^; Hispanic: OR = 1.40(1.19–1.64), *P* = 3.95*10^–5^); and reduced likelihood of receiving a prescription for clozapine (Black/AA: OR = 0.40(0.33–0.49), *P* = 2.16*10^–18^; Hispanic: OR = 0.45(0.35–0.58); *P* = 5.81*10^–10^), compared to white patients. For 23 of the 30 analyzed medications, the direction of the effect was consistent between Black/AA and Hispanic patients – indicating both groups were less/more likely to receive the medication compared to white patients, although many of these estimates were below the threshold for statistical significance after adjustment for multiple testing. Many of these effects were also observed in patients with unknown/’Other’ race/ethnicity (Fig. 1; Table S3), but not in Asian patients.

Several effects were observed uniquely in Hispanic patients, including higher likelihood of receiving a prescription for mirtazapine (OR = 1.70(1.3–2.23); *P* = 1.18*10^–4^) and trazodone (OR = 1.88(1.53–2.32); *P* = 3.54*10^–9^)—both atypical anti-depressants. Conversely, Black/AA patients were less likely to receive a prescription for several selective serotonin reuptake inhibitors (anti-depressants) than white patients (escitalopram: OR = 0.57(0.45–0.73)); *P* = 6.48*10^–6^; sertraline: OR = 0.69(0.57–0.85); *P* = 5.11*10^–4^; paroxetine: OR = 0.53(0.35–0.81); *P* = 3.07*10^–3^; lamotrigine(OR = 0.47 (0.33–0.66); *P* = 1.34*10^–5^).

Additional controlling for weight and BMI and exclusion of individuals with comorbid depression or BD (some of whom would be labeled as “schizoaffective” - see the Methods or our explanation of the comorbidity between schizophrenia and depression) did not affect the pattern of the results (Table S4).

**Fig. 2:**
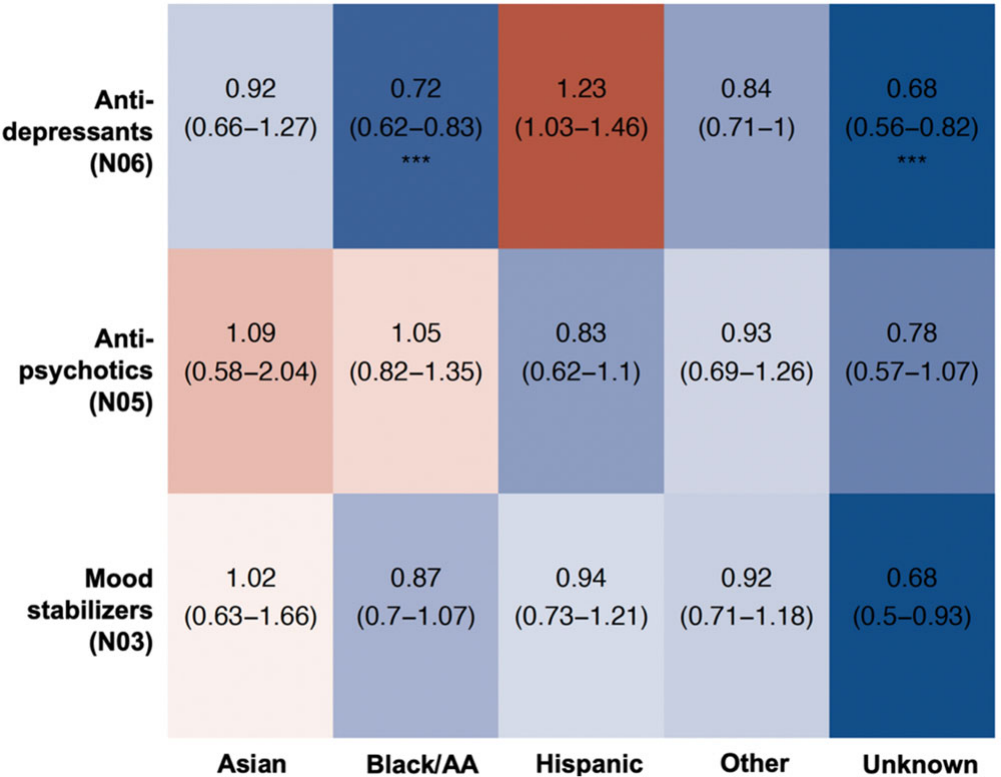
Likelihood of receiving an anti-psychotic, anti-depressant and mood stabilizing medication in Asian, Black/ AA, Hispanic, Other race/ethnicity and unknown race/ethnicity patients with SCZ diagnosis, compared to white patients. Red tiles indicate higher, blue tiles lower likelihood of receiving the prescription of the medication within each class; associated estimates reflect the odds ratios with their corresponding 95% confidence intervals. All analyses are adjusted for patient’s sex, age, comorbid depression and BD, weight and BMI, and account for the location of the provider. Multiple testing was accounted for using false discovery rate; *** Q-val <0.001; ** Q-val <0.01; * Q-val <0.05.

Analyzing the prescription data by therapeutic group, we observed that Black/AA patients and those with an unknown race/ethnicity were less likely to receive a prescription for anti-depressants than white patients, with no differences recorded for other races/ethnicities or therapeutic groups (Fig. 2)—suggesting that the differences observed on the individual medication level (Fig. 1) tended to cancel each other out within broader therapeutic group.

Performing equivalent analyses on patients diagnosed with BD or depression, we observed even more widespread differences in medication rates, including many of the effects recorded in the main (SCZ) analyses. For example, similarly to patients with SCZ, Hispanic and Black/AA patients with BD were more likely to receive haloperidol (Black/AA: OR = 1.69(1.48,1.92), *P* = 4.34*10^–15^; Hispanic: OR = 1.60(1.38–1.85), *P* = 2.03*10^–10^) and risperidone (Black/AA: OR = 1.77(1.58–2.00), *P* = 2.51*10^–21^; Hispanic: OR = 1.65(1.45–1.88), *P* = 1.86*10^–14^) than their white counterparts. Among the mood stabilizers (N03A), Black/AA and Hispanic patients with BD were significantly more likely to receive fluphenazine (Black/AA: OR = 1.72(1.20–2.45), *P* = 4.23*10^–3^; Hispanic: OR = 1.87(1.28–2.76), *P* = 2.31*10^–3^), chlorpromazine (Black/AA: OR = 1.76(1.34–2.32), *P* = 2.71*10^–5^; Hispanic: OR = 1.91(1.42–2.58), *P* = 8.80*10^–6^) and valproic acid (Black/AA: OR = 1.47(1.14–1.90), *P* = 3.38*10^–3^; Hispanic: OR = 1.51(1.14–2.00), *P* = 4.27*10^–3^), but less likely to receive lamotrigine (Black/AA: OR = 0.32(0.28–0.36), *P* = 4.73*10^–76^; Hispanic: OR = 0.48(0.43–0.54), *P* = 5.35*10^–34^; see Tables S5, S6 for results for BD and depression).

**Fig. 3:**
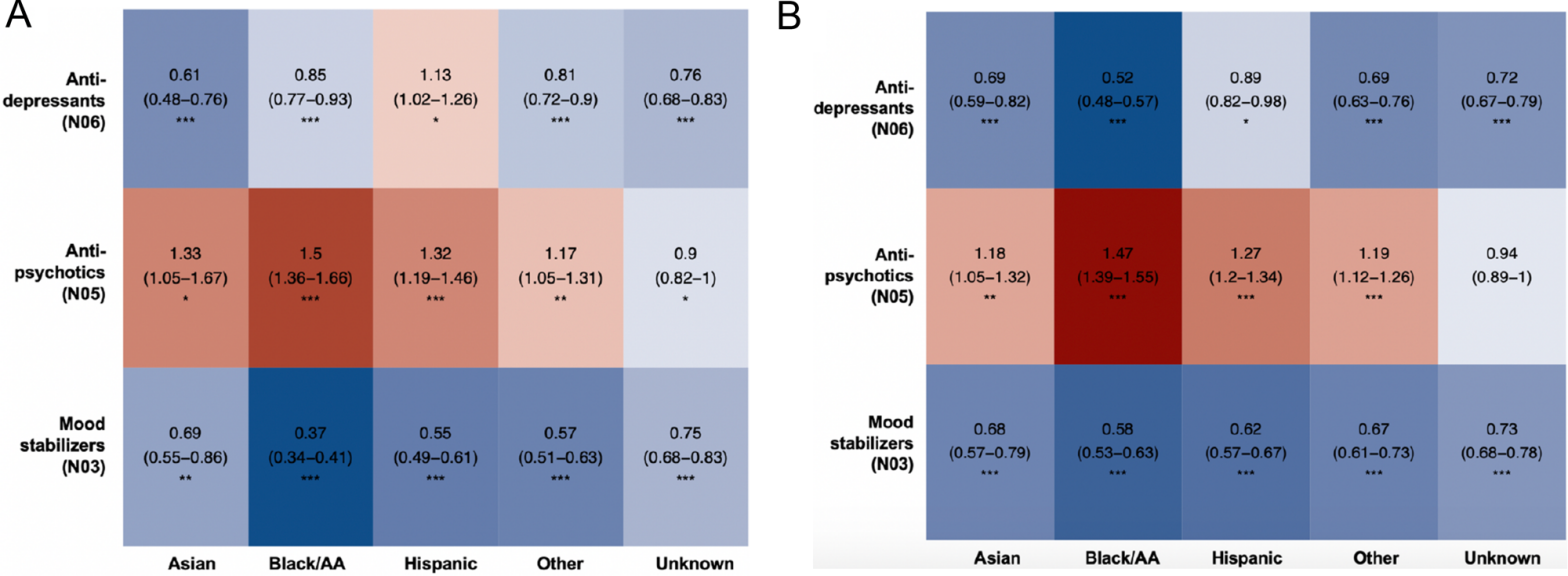
Likelihood of receiving an anti-psychotic, anti-depressant and mood stabilizing medication in Asian, Black/AA, Hispanic, Other race/ethnicity and unknown race/ethnicity patients with bipolar disorder and depression, compared to white patients. Red tiles indicate higher, blue tiles lower likelihood of receiving the prescription of the medication within each class among patients with BD (**A**) and depression (**B**) compared to white patients with those diagnoses; associated estimates reflect the odds ratios with their corresponding 95% confidence intervals. All analyses are adjusted for patient’s sex, age, comorbid SCZ and depression (**A**), BD (**B**), weight and BMI, and accounting for the location of the provider. Multiple testing was accounted for using false discovery rate; *** Q-val <0.001; ** Q-val <0.01; * Q-val <0.05.

Looking at the broader therapeutic categories, Asian, Hispanic and Black/AA patients with BD or depression were more likely to receive an anti-psychotic medication, but less likely to receive an anti-depressant or a mood stabilizer, compared to white patients (Fig. 3). The only exception to that pattern were Hispanic patients with BD, who were more likely to receive an anti-depressant compared to white patients.

### Medication dose

Among patients with SCZ, there were no differences in the prescribed maximum doses of medications by patient race/ethnicity (Table S7), including after additional controlling for patient weight and BMI, or excluding patients with comorbid BD or depression (Table S7).

**Fig. 4:**
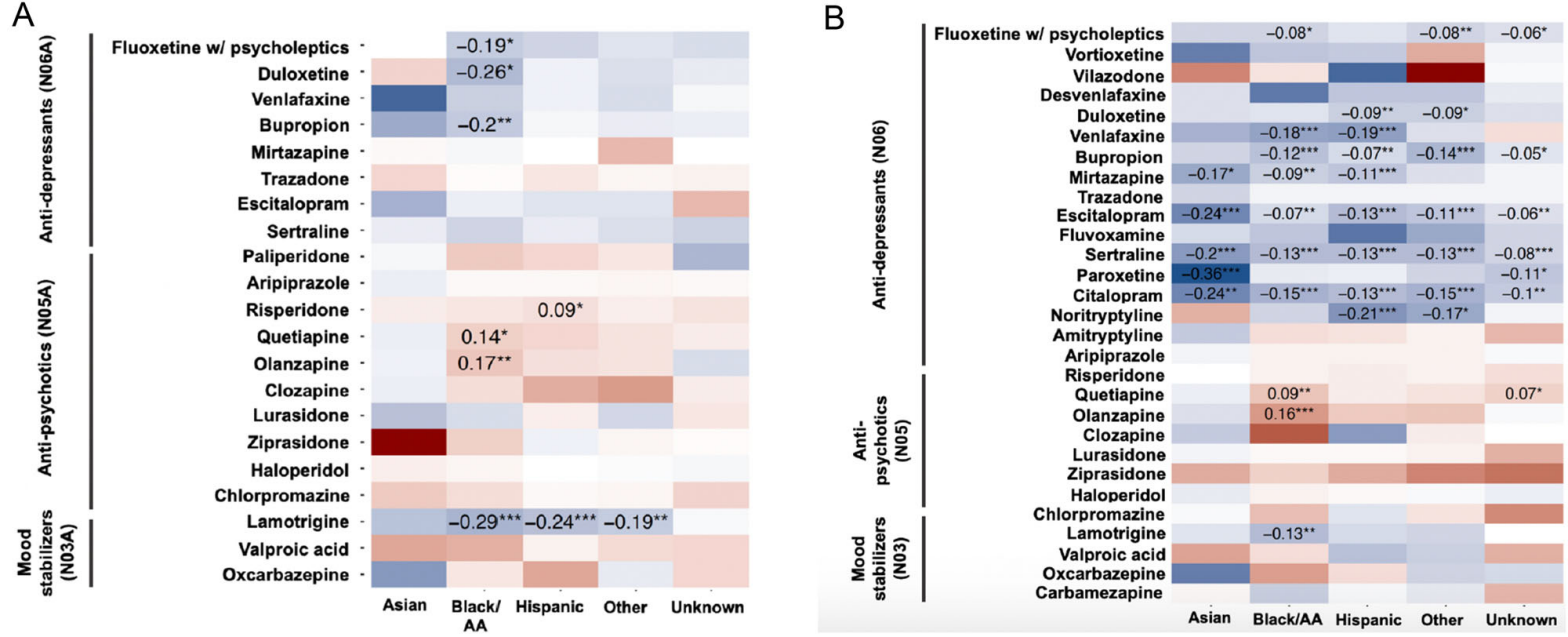
Standardized differences (Z-scores) in the doses of anti-psychotic, anti-depressant and mood stabilizing medications in Asian, Black/ AA, Hispanic, Other race/ethnicity and unknown race/ethnicity patients with bipolar disorder and depression, compared to white patients with respective diagnosis. Red tiles indicate higher, and blue tiles lower daily dose of the prescription of the given medication among patients with bipolar disorder (**A**) and depression (**B**), expressed as T-value. All analyses are adjusted for patient’s sex, age, comorbid depression and BD, and extended release status, and account for the location of the provider. Multiple testing was accounted for using false discovery rate; *** Q-val <0.001; ** Q-val <0.01; * Q-val <0.05.

However, we observed differences in the maximum doses of several medications prescribed to non-white patients with BD and depression (Fig. 4; Tables S8, S9). Those differences in medication doses followed the pattern observed for the medication rates, i.e., maximum doses were higher for several anti-psychotics, and lower for anti-depressants and mood stabilizers. These results indicate that Asian, Black/AA and Hispanic patients with BD or depression are more likely to receive an antipsychotic than white patients, and once they do so, the dose tends to be higher, with a converse pattern recorded for anti-depressants and mood stabilizers (i.e., lower rates and doses).

## Discussion

We found racial and ethnic differences in the medications prescribed to patients with SCZ, BD and depression, in a large-scale sample from one of the largest and most diverse hospital networks in New York. Our findings show that Black/AA and Hispanic patients with SCZ are more likely to be prescribed certain antipsychotics, most notably haloperidol and risperidone, and less likely to be prescribed clozapine, compared to white patients. In addition, Hispanic patients had a higher likelihood of being prescribed atypical antidepressants mirtazapine and trazadone, whereas Black/AA patients had a lower likelihood of being prescribed selective serotonin reuptake inhibitors (SSRIs) and other antidepressants. Furthermore, Black/AA and Hispanic patients were less likely to receive medications targeting mood symptoms. There were no differences in the prescribed doses of medications by patient race/ethnicity. These findings are consistent with previous studies^21–24^, and add new insights regarding the patterns of specific medications, differences in the dosage, and generalizability to other psychiatric conditions. Specifically, we have showed that Black/AA and Hispanic patients with BD and depression are more likely to be prescribed antipsychotics, but less likely to be prescribed antidepressants or mood stabilizers—and even once they are, they are prescribed at lower doses compared to white patients. Our results were robust to adjustments for a variety of potential confounders and to accounting for providers’ location—proxy for both neighborhood-level factors and distinct prescription practices across different locations.

Implications and reasons for these discrepancies require further inquiry, and the complex causes of the racial differences in the psychopharmacological treatment of psychiatric conditions could exist at patient-, provider- and systemic levels. These include patient preference for certain treatments, history of non-adherence, or concerns related to physical health. While these patient-level factors vary greatly across individuals, they can also be associated with patient’s race/ethnicity, e.g. higher levels of mistrust in the healthcare system among Black/AA patients^25^ in response to historical medical and scientific abuse of Black individuals^26,27^. On the systemic level, there is now a considerable body of research on unconscious bias within the healthcare system, which may influence healthcare professionals’ clinical judgments, treatment decisions, and communication with patients^28–31^, and result in suboptimal care, with wide-ranging, quality-of-life implications for ethnic minority patients. Acknowledging and addressing these issues within the healthcare system is crucial for ensuring that patients from all racial and ethnic backgrounds receive equitable, culturally competent, and effective mental health care. As our data came from numerous providers within a hospital network, we emphasize that these findings are unlikely to reflect biases specific to individual institutions, but likely generalize across healthcare settings.

Disparity in use of clozapine in Black/AA and Hispanic patients is of particular concern because of its efficacy for treatment-resistant SCZ – raising a possibility that these patients either may not be receiving the necessary treatment, or that, due to a potential over-diagnosis of SCZ in these populations^28^, their symptoms are less severe and less likely to require clozapine use. A potential reason underlying lower rates of clozapine prescription in Black/AA patients could be the requirement of regular leukocyte counts to monitor the risk of agranulocytosis. Research has suggested that physicians may believe that non-white patients are less compliant with medications and monitoring^29^. Additionally, benign ethnic neutropenia (BEN) among Black/AA patients—a naturally lower absolute neutrophil count (ANC) prevalent among individuals of sub-Saharan ancestry—historically rendered approximately 20% of Black/AA patients below the threshold (ANC > 1500/µL) for a clozapine prescription (NB. this does not apply to Hispanic patients)^30^. The consequences of applying clinical thresholds that are not normalized for ancestry have been recently highlighted for e.g. kidney function^31^, and similar effects may contribute to the patterns observed in our data. The guidelines for allowing clozapine’s use for ANCs <1500/µL in cases of baseline BEN were updated as of November 2021, however the patient data used in this study predates this revision.

Black/AA and Hispanic patients were more likely to receive haloperidol and risperidone prescriptions, both of which are available in oral and LAI formulations. While our analyses of the medication rates did not distinguish between the two forms, the elevated prescription rates of haloperidol and risperidone may be linked to an increased utilization of LAIs among these demographics. This explanation is in line with the previous studies showing that LAIs are predominantly recommended for patients perceived as non-compliant to oral antipsychotics, and are more likely to be given to individuals who tend to be younger, male, and Black^32,33^. Nevertheless, we lacked relevant information in our data to test this directly, and as such this interpretation remains speculative. Additionally, as LAI formulations are prescribed at higher doses^34^, lack of group differences in the Dose analyses does not support the LAI explanation. As both risperidone and haloperidol are prescribed more frequently as oral medications, rather than LAIs, the higher rate of haloperidol and risperidone prescriptions could be attributed to incomplete adjustment for the patient’s insurance status, rather than medication’s formulation. While racial and ethnic disparities in medication adherence may exist, they are often mediated by a complex interplay of factors beyond race—this includes clinical symptoms, side effects, individual patient characteristics^35^, SES, access to care, cultural beliefs, and quality of therapeutic relationship between patients and healthcare providers^36^. Understanding these nuances is crucial for developing targeted interventions to improve medication adherence, reduce disparities in mental healthcare outcomes across diverse populations and ensure that choice of medication and its formulation is not driven by perceived, but rather actual non-adherence.

Importantly, the differences in treatment by patient race/ethnicity did not appear to be specific to schizophrenia, but generalized to the other outcomes we tested – depression and bipolar disorder. Black/AA and Hispanic patients diagnosed with these conditions were less likely to be prescribed anti-depressants and mood stabilizers – medications that target the key symptoms of those disorders – but more likely to receive anti-psychotics, in comparison to their white counterparts. Additionally, Black/AA and Hispanic patients with depression and BD received anti-depressant and mood stabilizing medication at lower, and antipsychotic medication at higher doses compared to white patients. These results persisted after excluding patients with comorbid diagnoses, suggesting that our findings were not due to a higher rate of comorbidity in those populations. Studies support these findings, showing patterns of underutilization of antidepressants^37^ and mood stabilizers^38^ and overutilization of antipsychotics among Black/AA and Hispanic individuals with depression and BD^23^. Further research is needed to understand the underlying factors contributing to these disparities and develop interventions to mitigate them.

The strengths of this study include reliance on a diverse and large-scale sample, access to data on demographic and clinical factors, and accounting for the provider’s location – allowing us to conduct well-powered and comprehensive analyses, minimizing the effects of neighborhood-level factors or differences in prescription practices across settings. Ascertainment of individual medications allowed us to uncover specific medication patterns that might have been obscured in previous studies looking at the rates of anti-psychotics overall. Furthermore, included patients of multiple races/ethnicities, providing novel insights about the similarities and differences between these groups. Finally, we were both able to account for comorbid mood disorders, and interrogate whether the prescription patterns in patients with SCZ generalize to depression and BD.

There are a few limitations with this study that should be considered when interpreting our findings. Although we controlled for the location of the provider, practice variations may exist at the prescriber and patient level, including factors that cannot be accounted for in an EHR study (e.g. physician-patient rapport, patient preference). We did not differentiate between outpatient and inpatient care, and lacked data on patients’ psychiatric history, which precluded us from testing the influence of e.g. severity and duration of presenting symptoms, history of treatment/noncompliance, and information on patient’s physical comorbidities. Additionally, some of the medications considered in our analyses can be prescribed for diverse indications, and in the absence of clinical notes we were not able to verify the exact reason for the prescriptions. Further, although we used maximum dose prescribed in the Dose analyses, we could not account for how long the maximum dose was maintained. While we accounted for insurance status, studies have shown that socioeconomic status and insurance can be differentially associated with healthcare provision^39,40^. As the analyses of the prescription patterns among patients with BD and depression were performed primarily to provide broader context and enable us to gauge the extent to which differences in the prescriptions are specific to SCZ, racial and ethnic disparities in the treatment of those disorders warrants further, targeted interrogation. Lastly, the categorization of race in a medical record system may not consistently or precisely be measured, even when individuals are self-reporting. In our sample, 16% of patients were missing data on self-reported race and ethnicity, and 10% denoted an ‘Other’ race or ethnicity. Results for ‘Unknown’ and ‘Other’ race/ethnicity (Figs. 1–4) tended to follow the same trends as those found for Black/AA and Hispanic patients. The overlap in results suggests that the group of patients in the ‘Unknown’ and ‘Other’ categories primarily consisted of Black/AA and Hispanic patients. In part, this could be due to that fact that, for example, White-Hispanic and Black-Hispanic were not options the individuals could self-report. Patients of mixed race or who identify as more than one of the designated categories can thus be more likely to fall under the ‘Unknown’ and ‘Other’ demographics.

In conclusion, we found racial and ethnic disparities in the pharmacologic treatment of schizophrenia, bipolar disorder, and depression. The results of this study may implore providers to question prescription patterns in order to improve treatment practices and outcomes for all patients, and ensure adequate and equitable care. Future research is needed to understand the complex and heterogeneous causes of the patterns observed in our study.

### Supplementary information

Supplemental Tables

## Data Availability

Data supporting the findings of this study are available within the paper and in supplemental tables. We can share individual-level data only upon IRB approval, but will respond to ad hoc requests for data summaries.

## Code availability

All code is available upon request.
