# Supplementary material for: Differences in schizophrenia treatments by race and ethnicity—analysis of electronic health records
Source: Schizophrenia (Heidelb). 2024 Apr 26;10(1):48. doi: 10.1038/s41537-024-00470-4 (PMC11053048; doi:10.1038/s41537-024-00470-4)
Supplement: Supplementary file 1 — Supplemental Tables [file 41537_2024_470_MOESM1_ESM.docx]

## Supplemental material

|  | **White [ref]** | **Black / African American** | **Hispanic** | **Asian** | **American Indian or Alaska Native** | **Native Hawaiian or Pacific Islander** | **Other** | **Unknown** |
| --- | --- | --- | --- | --- | --- | --- | --- | --- |
| **N** | 7,574 | 3,332 | 2,594 | 384 | 9 | 12 | 2,253 | 2,597 |
| **% Female** | 54.9 | 60.4 | 61.0 | 64.1 | 66.7 | 58.3 | 57.2 | 59.7 |
| **Mean age (SD)** | 52.5 (17.8) | 48.0 (15.2) | 48.1  (16.0) | 42.1 (16.6) | 53.0 (16.9) | 43.9 (20.7) | 47.4 (17.1) | 48.3 (17.1) |
| **% depression** | 36.2 | 39.0 | 49.6 | 34.1 | 55.6 | 25.0 | 37.7 | 27.0 |
| **% schizophrenia** | 3.3 | 15.7 | 10.0 | 6.0 | 0 | 0 | 9.9 | 4.4 |
| **% private insurance** | 5.7 | 5.5 | 6.2 | 3.1 | 0 | 0 | 6.2 | 4.9 |
| **Mean BMI (SD)** | 26.4 (6.7) | 26.3  (6.6) | 26.1  (6.7) | 26.2 (6.8) | 19.0  (1.0) | 29.4  (0.4) | 26.0 (6.8) | 26.1  (6.8) |

**Table S1.** Characteristics of patients with a bipolar disorder diagnosis (ICD-10: F31).

|  | **White [ref]** | **Black / African American** | **Hispanic** | **Asian** | **American Indian or Alaska Native** | **Native Hawaiian or Pacific Islander** | **Other** | **Unknown** |
| --- | --- | --- | --- | --- | --- | --- | --- | --- |
| **N** | 59,180 | 13,278 | 17,203 | 3,104 | 88 | 83 | 12,292 | 20,515 |
| **% Female** | 65.4 | 66.6 | 70.4 | 68.1 | 72.7 | 74.7 | 64.5 | 69.6 |
| **Mean age (SD)** | 57.1 (19.8) | 53.6 (18.0) | 55.9 (18.3) | 48.1 (19.3) | 48.2 (17.6) | 50.7 (21.0) | 54.7 (19.9) | 52.0 (19.6) |
| **% schizophrenia** | 0.8 | 5.6 | 2.9 | 1.9 | 2.3 | 1.2 | 3.1 | 1.0 |
| **% bipolar disorder** | 4.6 | 9.8 | 7.5 | 4.2 | 5.7 | 3.6 | 6.9 | 3.4 |
| **% private insurance** | 6.0 | 5.0 | 5.7 | 8.3 | 10.2 | 1.2 | 8.0 | 5.3 |
| **Mean BMI (SD)** | 26.4 (6.8) | 26.5  (6.9) | 25.8  (6.8) | 26.5 (6.9) | 26.4  (6.0) | 29.1  (6.4) | 26.2 (6.9) | 26.5  (6.8) |

**Table S2.** Characteristics of patients with a depression diagnosis (ICD-10: F32-F33).

| **medication** | **ATC code** | **Race/ethnicity** | **OR (CI)** | **P-value** | **Q-value** |
| --- | --- | --- | --- | --- | --- |
| carbamazepine | N03AF01 | Black/African American | 1.6 (0.84-3.02) | 0.15 | 0.34 |
|  |  | Hispanic | 1.3 (0.6-2.8) | 0.51 | 0.72 |
|  |  | Other | 2.07 (1.03-4.14) | 0.04 | 0.14 |
|  |  | Unknown | 1.09 (0.46-2.6) | 0.84 | 0.91 |
| oxcarbazepine | N03F02 | Black/African American | 0.88 (0.53-1.48) | 0.63 | 0.79 |
|  |  | Hispanic | 0.9 (0.48-1.68) | 0.73 | 0.84 |
|  |  | Other | 1.16 (0.65-2.08) | 0.62 | 0.78 |
|  |  | Unknown | 0.68 (0.32-1.43) | 0.31 | 0.54 |
| valproic acid | N03AG01 | Black/African American | 1.1 (0.82-1.46) | 0.53 | 0.73 |
|  |  | Hispanic | 1.21 (0.86-1.7) | 0.28 | 0.52 |
|  |  | Other | 1.11 (0.79-1.55) | 0.55 | 0.73 |
|  |  | Unknown | 0.89 (0.59-1.35) | 0.59 | 0.75 |
|  |  | Asian | 0.94 (0.47-1.87) | 0.86 | 0.91 |
| lamotrigine | N03AX09 | Black/African American | 0.47 (0.33-0.66) | 1.34E-05 | 2.27E-04 |
|  |  | Hispanic | 0.65 (0.44-0.96) | 0.03 | 0.11 |
|  |  | Other | 0.45 (0.29-0.7) | 3.7E-04 | 4.43E-03 |
|  |  | Unknown | 0.44 (0.27-0.74) | 1.75E-03 | 0.01 |
|  |  | Asian | 0.91 (0.46-1.81) | 0.79 | 0.86 |
| chlorpromazine | N05AA01 | Black/African American | 0.91 (0.66-1.24) | 0.55 | 0.73 |
|  |  | Hispanic | 1.24 (0.86-1.78) | 0.24 | 0.46 |
|  |  | Other | 0.84 (0.58-1.2) | 0.33 | 0.58 |
|  |  | Unknown | 0.59 (0.36-0.95) | 0.03 | 0.11 |
| fluphenazine | N05AB02 | Black/African American | 0.96 (0.77-1.2) | 0.75 | 0.85 |
|  |  | Hispanic | 0.88 (0.67-1.16) | 0.35 | 0.59 |
|  |  | Other | 0.87 (0.67-1.13) | 0.30 | 0.54 |
|  |  | Unknown | 0.93 (0.7-1.25) | 0.65 | 0.79 |
|  |  | Asian | 0.7 (0.4-1.22) | 0.21 | 0.42 |
| perphenazine | N05AB03 | Black/African American | 0.74 (0.52-1.05) | 0.09 | 0.26 |
|  |  | Hispanic | 0.68 (0.43-1.06) | 0.09 | 0.26 |
|  |  | Other | 1 (0.67-1.48) | 0.98 | 0.99 |
|  |  | Unknown | 0.73 (0.46-1.15) | 0.17 | 0.37 |
| trifluoperazine | N05AB06 | Black/African American | 0.82 (0.34-1.97) | 0.65 | 0.79 |
|  |  | Hispanic | 0.36 (0.08-1.65) | 0.19 | 0.38 |
|  |  | Other | 0.33 (0.07-1.53) | 0.16 | 0.35 |
|  |  | Unknown | 2.29 (0.99-5.32) | 0.05 | 0.17 |
| haloperidol | N05AD01 | Black/African American | 1.52 (1.33-1.74) | 0.00 | 0.00 |
|  |  | Hispanic | 1.32 (1.12-1.56) | 0.00 | 0.01 |
|  |  | Other | 1.32 (1.12-1.55) | 0.00 | 0.01 |
|  |  | Unknown | 0.91 (0.75-1.09) | 0.30 | 0.54 |
|  |  | Asian | 0.62 (0.45-0.86) | 0.00 | 0.02 |
| ziprasidone | N05AE04 | Black/African American | 0.78 (0.57-1.07) | 0.13 | 0.30 |
|  |  | Hispanic | 0.93 (0.64-1.35) | 0.71 | 0.84 |
|  |  | Other | 0.86 (0.59-1.25) | 0.43 | 0.65 |
|  |  | Unknown | 0.78 (0.51-1.18) | 0.24 | 0.45 |
| lurasidone | N05AE05 | Black/African American | 0.54 (0.38-0.77) | 0.00 | 0.01 |
|  |  | Hispanic | 0.73 (0.49-1.09) | 0.12 | 0.30 |
|  |  | Other | 0.56 (0.36-0.86) | 0.01 | 0.04 |
|  |  | Unknown | 0.45 (0.26-0.77) | 0.00 | 0.02 |
| clozapine | N05AH02 | Black/African American | 0.4 (0.33-0.49) | 0.00 | 0.00 |
|  |  | Hispanic | 0.45 (0.35-0.58) | 0.00 | 0.00 |
|  |  | Other | 0.54 (0.43-0.69) | 0.00 | 0.00 |
|  |  | Unknown | 0.54 (0.41-0.71) | 0.00 | 0.00 |
|  |  | Asian | 0.84 (0.46-1.81) | 0.36 | 0.59 |
| olanzapine | N05AH03 | Black/African American | 0.85 (0.74-0.98) | 0.03 | 0.11 |
|  |  | Hispanic | 0.98 (0.83-1.16) | 0.82 | 0.89 |
|  |  | Other | 0.91 (0.77-1.07) | 0.24 | 0.45 |
|  |  | Unknown | 0.75 (0.63-0.91) | 0.00 | 0.02 |
|  |  | Asian | 1.29 (0.96-1.71) | 0.09 | 0.26 |
| quetiapine | N05AH04 | Black/African American | 0.96 (0.82-1.13) | 0.65 | 0.79 |
|  |  | Hispanic | 1.3 (1.08-1.56) | 0.01 | 0.03 |
|  |  | Other | 1.02 (0.84-1.23) | 0.85 | 0.91 |
|  |  | Unknown | 0.88 (0.71-1.09) | 0.23 | 0.45 |
|  |  | Asian | 0.87 (0.6-1.20) | 0.49 | 0.72 |
| risperidone | N05AX08 | Black/African American | 1.27 (1.11-1.45) | 0.00 | 0.01 |
|  |  | Hispanic | 1.4 (1.19-1.64) | 0.00 | 0.00 |
|  |  | Other | 1.13 (0.97-1.33) | 0.12 | 0.30 |
|  |  | Unknown | 1.11 (0.93-1.32) | 0.26 | 0.49 |
|  |  | Asian | 1.28 (0.96-1.71) | 0.09 | 0.26 |
| aripiprazole | N05AX12 | Black/African American | 0.89 (0.76-1.05) | 0.15 | 0.35 |
|  |  | Hispanic | 1.09 (0.91-1.32) | 0.35 | 0.59 |
|  |  | Other | 1.03 (0.86-1.25) | 0.73 | 0.84 |
|  |  | Unknown | 0.84 (0.68-1.05) | 0.12 | 0.30 |
|  |  | Asian | 1.14 (0.74-1.77) | 0.43 | 0.65 |
| paliperidone | N05AX13 | Black/African American | 1.28 (1.03-1.6) | 0.03 | 0.11 |
|  |  | Hispanic | 1.32 (1.01-1.71) | 0.04 | 0.14 |
|  |  | Other | 1.08 (0.83-1.4) | 0.56 | 0.73 |
|  |  | Unknown | 0.86 (0.63-1.19) | 0.36 | 0.59 |
|  |  | Asian | 1.14 (0.4-1.22) | 0.55 | 0.73 |
| amitryptiline | N06AA09 | Black/African American | 1.09 (0.6-1.96) | 0.78 | 0.86 |
|  |  | Hispanic | 2.14 (1.19-3.85) | 0.01 | 0.06 |
|  |  | Other | 0.88 (0.42-1.82) | 0.73 | 0.84 |
|  |  | Unknown | 0.98 (0.43-2.23) | 0.97 | 0.99 |
| nortriptyline | N06AA10 | Black/African American | 0.7 (0.33-1.48) | 0.35 | 0.59 |
|  |  | Hispanic | 0.87 (0.38-2.02) | 0.75 | 0.85 |
|  |  | Other | 0.34 (0.11-1.1) | 0.07 | 0.21 |
|  |  | Unknown | 0.27 (0.06-1.26) | 0.10 | 0.26 |
| citalopram | N06AB04 | Black/African American | 0.82 (0.63-1.06) | 0.13 | 0.32 |
|  |  | Hispanic | 1.11 (0.82-1.49) | 0.50 | 0.72 |
|  |  | Other | 1.01 (0.75-1.36) | 0.96 | 0.99 |
|  |  | Unknown | 0.88 (0.62-1.25) | 0.46 | 0.69 |
| paroxetine | N06AB05 | Black/African American | 0.53 (0.35-0.81) | 0.00 | 0.02 |
|  |  | Hispanic | 0.83 (0.53-1.31) | 0.43 | 0.65 |
|  |  | Other | 0.96 (0.62-1.49) | 0.87 | 0.91 |
|  |  | Unknown | 0.59 (0.33-1.03) | 0.06 | 0.20 |
| sertraline | N06AB06 | Black/African American | 0.69 (0.57-0.85) | 0.00 | 0.01 |
|  |  | Hispanic | 1.09 (0.87-1.37) | 0.44 | 0.66 |
|  |  | Other | 0.9 (0.71-1.14) | 0.37 | 0.60 |
|  |  | Unknown | 0.6 (0.45-0.8) | 0.00 | 0.01 |
|  |  | Asian | 0.73 (0.46-1.18) | 0.20 | 0.40 |
| fluvoxamine | N06AB08 | Black/African American | 0.35 (0.13-0.92) | 0.03 | 0.12 |
|  |  | Hispanic | 0.75 (0.27-2.05) | 0.57 | 0.74 |
|  |  | Other | 0.48 (0.17-1.4) | 0.18 | 0.37 |
|  |  | Unknown | 0.08 (0.01-0.77) | 0.03 | 0.11 |
| escitalopram | N06AB10 | Black/African American | 0.57 (0.45-0.73) | 0.00 | 0.00 |
|  |  | Hispanic | 0.74 (0.56-0.97) | 0.03 | 0.11 |
|  |  | Other | 0.63 (0.47-0.84) | 0.00 | 0.01 |
|  |  | Unknown | 0.56 (0.4-0.78) | 0.00 | 0.01 |
|  |  | Asian | 1.22 (0.77-1.93) | 0.40 | 0.63 |
| trazodone | N06AX05 | Black/African American | 1.18 (0.98-1.43) | 0.08 | 0.24 |
|  |  | Hispanic | 1.88 (1.53-2.32) | 0.00 | 0.00 |
|  |  | Other | 1.28 (1.03-1.59) | 0.03 | 0.11 |
|  |  | Unknown | 0.92 (0.7-1.19) | 0.51 | 0.72 |
|  |  | Asian | 1 (0.64-1.56) | 0.99 | 0.99 |
| mirtazapine | N06AX11 | Black/African American | 1.05 (0.82-1.35) | 0.71 | 0.84 |
|  |  | Hispanic | 1.7 (1.3-2.23) | 0.00 | 0.00 |
|  |  | Other | 1.37 (1.03-1.83) | 0.03 | 0.11 |
|  |  | Unknown | 0.95 (0.67-1.33) | 0.75 | 0.85 |
|  |  | Asian | 0.85 (0.45-0.86) | 0.64 | 0.79 |
| bupropion | N06AX12 | Black/African American | 0.84 (0.66-1.07) | 0.16 | 0.36 |
|  |  | Hispanic | 0.91 (0.69-1.21) | 0.52 | 0.72 |
|  |  | Other | 0.79 (0.58-1.06) | 0.12 | 0.30 |
|  |  | Unknown | 0.76 (0.54-1.07) | 0.12 | 0.30 |
| venlafaxine | N06AX16 | Black/African American | 0.56 (0.38-0.81) | 0.00 | 0.02 |
|  |  | Hispanic | 1.03 (0.7-1.52) | 0.90 | 0.94 |
|  |  | Other | 0.82 (0.54-1.24) | 0.35 | 0.59 |
|  |  | Unknown | 0.92 (0.58-1.46) | 0.71 | 0.84 |
| duloxetine | N06AX21 | Black/African American | 0.88 (0.61-1.28) | 0.51 | 0.72 |
|  |  | Hispanic | 1 (0.66-1.53) | 0.99 | 0.99 |
|  |  | Other | 0.99 (0.64-1.54) | 0.98 | 0.99 |
|  |  | Unknown | 0.68 (0.39-1.19) | 0.18 | 0.37 |
|  |  | Asian | 2.18 (1.11-4.29) | 0.02 | 0.11 |
| fluoxetine and psycholeptics | N06CA03 | Black/African American | 0.77 (0.6-1) | 0.05 | 0.17 |
|  |  | Hispanic | 0.91 (0.68-1.23) | 0.55 | 0.73 |
|  |  | Other | 0.88 (0.65-1.19) | 0.40 | 0.63 |
|  |  | Unknown | 0.72 (0.51-1.03) | 0.07 | 0.21 |
|  |  | Asian | 1.08 (0.64-1.83) | 0.77 | 0.85 |

**Table S3.** Association between race/ethnicity and receiving a prescription of anti-psychotic, anti-depressant and anti-epileptic medications in MSHS among schizophrenia patients. All analyses are adjusted for patient sex, age, comorbid depression and bipolar disorder, and account for the location of the provider. Multiple testing was accounted for using false discovery rate (Q-value)

| **medication** | **ATC code** | **Race /**  **ethnicity** | **OR (CI),**  **main**  **(N=9,104)** | **OR (CI),**  **weight + BMI (N=3,650)** | **OR (CI),**  **no comorbidity (N=7,581)** |
| --- | --- | --- | --- | --- | --- |
| carbamazepine | N03AF01 | Black/African American | 1.60 (0.84-3.02) | 1.71 (0.88-3.32) | 1.32 (0.59-2.92) |
|  |  | Hispanic | 1.30 (0.6-2.8) | 1.43 (0.64-3.2) | 0.43 (0.11-1.72) |
|  |  | Other | 2.07 (1.03-4.14) | 2.26 (1.08-4.7) | 0.74 (0.26-2.14) |
|  |  | Unknown | 1.09 (0.46-2.6) | 1.24 (0.5-3.05) | 0.73 (0.24-2.22) |
| oxcarbazepine | N03AF02 | Black/African American | 0.88 (0.53-1.48) | 0.82 (0.49-1.38) | 0.66 (0.33-1.34) |
|  |  | Hispanic | 0.9 (0.48-1.68) | 0.81 (0.43-1.52) | 0.48 (0.17-1.38) |
|  |  | Other | 1.16 (0.65-2.08) | 0.92 (0.49-1.71) | 1.19 (0.56-2.54) |
|  |  | Unknown | 0.68 (0.32-1.43) | 0.59 (0.26-1.33) | 0.54 (0.2-1.45) |
| valproic acid | N03AG01 | Black/African American | 1.1 (0.82-1.46) | 1.17 (0.86-1.6) | 0.96 (0.67-1.39) |
|  |  | Hispanic | 1.21 (0.86-1.7) | 1.34 (0.93-1.93) | 0.99 (0.62-1.58) |
|  |  | Other | 1.11 (0.79-1.55) | 1.24 (0.86-1.78) | 1.09 (0.72-1.66) |
|  |  | Unknown | 0.89 (0.59-1.35) | 0.94 (0.6-1.49) | 0.84 (0.51-1.38) |
|  |  | Asian | 0.94 (0.47-1.87) |  | 0.92 (0.35-2.44) |
| lamotrigine | N03AX09 | Black/African American | 0.47 (0.33-0.66) | 0.48 (0.33-0.69) | 0.39 (0.22-0.68) |
|  |  | Hispanic | 0.65 (0.44-0.96) | 0.56 (0.36-0.85) | 0.63 (0.32-1.24) |
|  |  | Other | 0.45 (0.29-0.7) | 0.44 (0.27-0.7) | 0.50 (0.25-0.97) |
|  |  | Unknown | 0.44 (0.27-0.74) | 0.43 (0.24-0.76) | 0.62 (0.32-1.2) |
|  |  | Asian | 0.91 (0.46-1.81) | 0.91 (0.46-1.81) | 0.80 (NA-NA) |
| chlorpromazine | N05AA01 | Black/African American | 0.91 (0.66-1.24) | 0.89 (0.63-1.24) | 0.93 (0.62-1.4) |
|  |  | Hispanic | 1.24 (0.86-1.78) | 1.2 (0.82-1.77) | 1.25 (0.76-2.05) |
|  |  | Other | 0.84 (0.58-1.2) | 0.76 (0.51-1.14) | 0.93 (0.58-1.49) |
|  |  | Unknown | 0.59 (0.36-0.95) | 0.5 (0.28-0.87) | 0.66 (0.37-1.17) |
| fluphenazine | N05AB02 | Black/African American | 0.96 (0.77-1.2) | 0.96 (0.76-1.21) | 0.95 (0.73-1.24) |
|  |  | Hispanic | 0.88 (0.67-1.16) | 0.82 (0.61-1.1) | 0.87 (0.61-1.24) |
|  |  | Other | 0.87 (0.67-1.13) | 0.87 (0.65-1.15) | 0.79 (0.57-1.11) |
|  |  | Unknown | 0.93 (0.7-1.25) | 0.91 (0.66-1.25) | 1.00 (0.72-1.4) |
|  |  | Asian | 0.7 (0.4-1.22) | 0.7 (0.4-1.22) | 0.66 (NA-NA) |
| perphenazine | N05AB03 | Black/African American | 0.74 (0.52-1.05) | 0.75 (0.51-1.11) | 0.80 (0.52-1.25) |
|  |  | Hispanic | 0.68 (0.43-1.06) | 0.7 (0.43-1.14) | 0.73 (0.4-1.34) |
|  |  | Other | 1.00 (0.67-1.48) | 1.07 (0.69-1.65) | 1.2 (0.73-1.97) |
|  |  | Unknown | 0.73 (0.46-1.15) | 0.79 (0.48-1.32) | 0.75 (0.43-1.3) |
| trifluoperazine | N05AB06 | Black/African American | 0.82 (0.34-1.97) | 0.89 (0.35-2.24) | 0.6 (0.21-1.71) |
|  |  | Hispanic | 0.36 (0.08-1.65) | 0.39 (0.08-1.83) | 0 (0-Inf) |
|  |  | Other | 0.33 (0.07-1.53) | 0.4 (0.08-1.85) | 0.21 (0.03-1.67) |
|  |  | Unknown | 2.29 (0.99-5.32) | 2.41 (0.98-5.95) | 2.42 (0.98-5.97) |
| haloperidol | N05AD01 | Black/African American | 1.52 (1.33-1.74) | 1.53 (1.32-1.77) | 1.72 (1.45-2.04) |
|  |  | Hispanic | 1.32 (1.12-1.56) | 1.29 (1.08-1.54) | 1.46 (1.18-1.81) |
|  |  | Other | 1.32 (1.12-1.55) | 1.37 (1.15-1.64) | 1.31 (1.07-1.61) |
|  |  | Unknown | 0.91 (0.75-1.09) | 0.92 (0.75-1.13) | 0.97 (0.78-1.22) |
|  |  | Asian | 0.62 (0.45-0.86) | 0.62 (0.45-0.86) | 0.86 (NA-NA) |
| ziprasidone | N05AE04 | Black/African American | 0.78 (0.57-1.07) | 0.81 (0.58-1.14) | 0.79 (0.5-1.25) |
|  |  | Hispanic | 0.93 (0.64-1.35) | 0.9 (0.61-1.34) | 1.18 (0.69-2.03) |
|  |  | Other | 0.86 (0.59-1.25) | 0.95 (0.64-1.41) | 1.3 (0.78-2.14) |
|  |  | Unknown | 0.78 (0.51-1.18) | 0.85 (0.54-1.34) | 0.97 (0.57-1.67) |
| lurasidone | N05AE05 | Black/African American | 0.54 (0.38-0.77) | 0.47 (0.32-0.7) | 0.76 (0.44-1.31) |
|  |  | Hispanic | 0.73 (0.49-1.09) | 0.66 (0.43-1.02) | 1.16 (0.61-2.18) |
|  |  | Other | 0.56 (0.36-0.86) | 0.53 (0.33-0.85) | 0.57 (0.28-1.18) |
|  |  | Unknown | 0.45 (0.26-0.77) | 0.41 (0.22-0.76) | 0.60 (0.28-1.29) |
| clozapine | N05AH02 | Black/African American | 0.4 (0.33-0.49) | 0.39 (0.31-0.49) | 0.39 (0.3-0.5) |
|  |  | Hispanic | 0.45 (0.35-0.58) | 0.45 (0.34-0.58) | 0.45 (0.32-0.62) |
|  |  | Other | 0.54 (0.43-0.69) | 0.55 (0.43-0.71) | 0.60 (0.45-0.79) |
|  |  | Unknown | 0.54 (0.41-0.71) | 0.47 (0.34-0.64) | 0.57 (0.42-0.77) |
|  |  | Asian | 0.84 (0.46-1.81) | 0.84 (0.57-1.23) | 1.31 (0.43-1.51) |
| olanzapine | N05AH03 | Black/African American | 0.85 (0.74-0.98) | 0.86 (0.74-1) | 0.88 (0.74-1.05) |
|  |  | Hispanic | 0.98 (0.83-1.16) | 0.98 (0.82-1.18) | 0.88 (0.7-1.1) |
|  |  | Other | 0.91 (0.77-1.07) | 0.92 (0.77-1.1) | 0.86 (0.7-1.05) |
|  |  | Unknown | 0.75 (0.63-0.91) | 0.8 (0.65-0.98) | 0.82 (0.66-1.02) |
|  |  | Asian | 1.29 (0.96-1.73) | 1.29 (0.4-1.22) | 1.21 (NA-NA) |
| quetiapine | N05AH04 | Black/African American | 0.96 (0.82-1.13) | 0.95 (0.81-1.13) | 0.93 (0.75-1.15) |
|  |  | Hispanic | 1.3 (1.08-1.56) | 1.31 (1.08-1.59) | 1.33 (1.02-1.72) |
|  |  | Other | 1.02 (0.84-1.23) | 1.01 (0.83-1.24) | 1.07 (0.83-1.38) |
|  |  | Unknown | 0.88 (0.71-1.09) | 0.79 (0.62-0.99) | 0.96 (0.73-1.25) |
|  |  | Asian | 0.87 (0.6-1.28) | 0.87 (0.6-1.28) |  |
| risperidone | N05AX08 | Black/African American | 1.27 (1.11-1.45) | 1.32 (1.14-1.52) | 1.19 (1.01-1.41) |
|  |  | Hispanic | 1.4 (1.19-1.64) | 1.42 (1.2-1.69) | 1.35 (1.09-1.67) |
|  |  | Other | 1.13 (0.97-1.33) | 1.18 (1-1.4) | 1.06 (0.87-1.3) |
|  |  | Unknown | 1.11 (0.93-1.32) | 1.1 (0.91-1.34) | 0.98 (0.79-1.21) |
|  |  | Asian | 1.28 (0.96-1.71) | 1.28 (0.96-1.71) | 1.19 (NA-NA) |
| aripiprazole | N05AX12 | Black/African American | 0.89 (0.76-1.05) | 0.86 (0.72-1.02) | 0.90 (0.72-1.12) |
|  |  | Hispanic | 1.09 (0.91-1.32) | 1.08 (0.88-1.32) | 1.05 (0.8-1.38) |
|  |  | Other | 1.03 (0.86-1.25) | 1.12 (0.92-1.37) | 0.96 (0.74-1.24) |
|  |  | Unknown | 0.84 (0.68-1.05) | 0.95 (0.75-1.2) | 0.95 (0.72-1.25) |
|  |  | Asian | 1.14 (0.74-1.77) | 1.14 (0.82-1.6) | 1.03 (NA-NA) |
| paliperidone | N05AX13 | Black/African American | 1.28 (1.03-1.6) | 1.27 (1-1.63) | 1.28 (0.97-1.68) |
|  |  | Hispanic | 1.32 (1.01-1.71) | 1.41 (1.06-1.87) | 1.36 (0.98-1.89) |
|  |  | Other | 1.08 (0.83-1.4) | 1.05 (0.78-1.4) | 1.13 (0.82-1.55) |
|  |  | Unknown | 0.86 (0.63-1.19) | 0.76 (0.53-1.11) | 0.82 (0.56-1.2) |
|  |  | Asian | 1.14 (0.4-1.22) | 1.14 (0.74-1.77) | 0.76 (0.44-0.97) |
| amitryptiline | N06AA09 | Black/African American | 1.09 (0.6-1.96) | 1.11 (0.6-2.05) | 0.37 (0.14-1) |
|  |  | Hispanic | 2.14 (1.19-3.85) | 2 (1.09-3.68) | 0.63 (0.19-2.07) |
|  |  | Other | 0.88 (0.42-1.82) | 0.89 (0.42-1.9) | 0.38 (0.1-1.41) |
|  |  | Unknown | 0.98 (0.43-2.23) | 0.91 (0.37-2.25) | 0.28 (0.06-1.29) |
| nortriptyline | N06AA10 | Black/African American | 0.7 (0.33-1.48) | 0.76 (0.35-1.66) | 0.65 (0.18-2.31) |
|  |  | Hispanic | 0.87 (0.38-2.02) | 0.86 (0.35-2.1) | 1.26 (0.29-5.44) |
|  |  | Other | 0.34 (0.11-1.1) | 0.39 (0.12-1.28) | 0.61 (0.12-3.21) |
|  |  | Unknown | 0.27 (0.06-1.26) | 0.08 (0-1.55) | 0 (0-Inf) |
| citalopram | N06AB04 | Black/African American | 0.82 (0.63-1.06) | 0.87 (0.65-1.15) | 0.52 (0.35-0.77) |
|  |  | Hispanic | 1.11 (0.82-1.49) | 1.12 (0.82-1.54) | 1.21 (0.78-1.88) |
|  |  | Other | 1.01 (0.75-1.36) | 1.05 (0.75-1.45) | 0.81 (0.52-1.27) |
|  |  | Unknown | 0.88 (0.62-1.25) | 0.83 (0.55-1.23) | 0.96 (0.61-1.51) |
| paroxetine | N06AB05 | Black/African American | 0.53 (0.35-0.81) | 0.55 (0.36-0.85) | 0.46 (0.25-0.82) |
|  |  | Hispanic | 0.83 (0.53-1.31) | 0.85 (0.53-1.36) | 0.37 (0.15-0.92) |
|  |  | Other | 0.96 (0.62-1.49) | 1.04 (0.66-1.64) | 0.83 (0.44-1.57) |
|  |  | Unknown | 0.59 (0.33-1.03) | 0.62 (0.34-1.13) | 0.37 (0.16-0.84) |
| sertraline | N06AB06 | Black/African American | 0.69 (0.57-0.85) | 0.72 (0.58-0.89) | 0.67 (0.5-0.91) |
|  |  | Hispanic | 1.09 (0.87-1.37) | 1.12 (0.89-1.42) | 1.00 (0.69-1.45) |
|  |  | Other | 0.9 (0.71-1.14) | 0.88 (0.69-1.13) | 0.90 (0.64-1.28) |
|  |  | Unknown | 0.6 (0.45-0.8) | 0.63 (0.46-0.86) | 0.66 (0.45-0.98) |
|  |  | Asian | 0.73 (0.46-1.18) | 0.73 (0.45-0.86) | 0.89 (NA-NA) |
| fluvoxamine | N06AB08 | Black/African American | 0.35 (0.13-0.92) | 0.17 (0.05-0.53) | 0.19 (0.04-0.85) |
|  |  | Hispanic | 0.75 (0.27-2.05) | 0.76 (0.29-1.96) | 1.19 (0.36-4) |
|  |  | Other | 0.48 (0.17-1.4) | 0.49 (0.17-1.44) | 0.77 (0.21-2.8) |
|  |  | Unknown | 0.08 (0.01-0.77) | 0.11 (0.01-1.15) | 0.38 (0.07-2.07) |
| escitalopram | N06AB10 | Black/African American | 0.57 (0.45-0.73) | 0.54 (0.42-0.7) | 0.49 (0.34-0.71) |
|  |  | Hispanic | 0.74 (0.56-0.97) | 0.72 (0.54-0.96) | 0.75 (0.48-1.18) |
|  |  | Other | 0.63 (0.47-0.84) | 0.62 (0.46-0.84) | 0.48 (0.3-0.77) |
|  |  | Unknown | 0.56 (0.4-0.78) | 0.54 (0.37-0.78) | 0.46 (0.28-0.75) |
|  |  | Asian | 1.22 (0.77-1.93) | 1.22 (0.77-1.93) | 1.09 (NA-NA) |
| trazodone | N06AX05 | Black/African American | 1.18 (0.98-1.43) | 1.25 (1.02-1.53) | 1.36 (1.04-1.77) |
|  |  | Hispanic | 1.88 (1.53-2.32) | 1.9 (1.52-2.37) | 1.95 (1.43-2.67) |
|  |  | Other | 1.28 (1.03-1.59) | 1.39 (1.1-1.75) | 1.27 (0.92-1.74) |
|  |  | Unknown | 0.92 (0.7-1.19) | 0.94 (0.7-1.25) | 0.91 (0.64-1.3) |
|  |  | Asian | 1 (0.64-1.56) | 1 (0.64-1.56) | 4.39 (NA-NA) |
| mirtazapine | N06AX11 | Black/African American | 1.05 (0.82-1.35) | 1.11 (0.85-1.44) | 0.93 (0.64-1.36) |
|  |  | Hispanic | 1.7 (1.3-2.23) | 1.73 (1.3-2.28) | 1.7 (1.11-2.62) |
|  |  | Other | 1.37 (1.03-1.83) | 1.39 (1.04-1.88) | 1.07 (0.68-1.67) |
|  |  | Unknown | 0.95 (0.67-1.33) | 0.91 (0.63-1.32) | 0.55 (0.32-0.95) |
|  |  | Asian | 0.85 (0.45-0.86) |  | 0.78 (0.55-1.36) |
| bupropion | N06AX12 | Black/African American | 0.84 (0.66-1.07) | 0.83 (0.64-1.08) | 0.72 (0.49-1.06) |
|  |  | Hispanic | 0.91 (0.69-1.21) | 0.95 (0.7-1.28) | 1.01 (0.64-1.6) |
|  |  | Other | 0.79 (0.58-1.06) | 0.85 (0.62-1.17) | 0.86 (0.54-1.35) |
|  |  | Unknown | 0.76 (0.54-1.07) | 0.85 (0.59-1.22) | 0.76 (0.47-1.22) |
| venlafaxine | N06AX16 | Black/African American | 0.56 (0.38-0.81) | 0.5 (0.34-0.75) | 0.34 (0.19-0.62) |
|  |  | Hispanic | 1.03 (0.7-1.52) | 0.92 (0.61-1.4) | 0.77 (0.39-1.51) |
|  |  | Other | 0.82 (0.54-1.24) | 0.81 (0.52-1.25) | 0.72 (0.38-1.34) |
|  |  | Unknown | 0.92 (0.58-1.46) | 1.02 (0.63-1.64) | 0.76 (0.4-1.45) |
| duloxetine | N06AX21 | Black/African American | 0.88 (0.61-1.28) | 0.88 (0.59-1.33) | 0.78 (0.41-1.5) |
|  |  | Hispanic | 1 (0.66-1.53) | 1.01 (0.64-1.6) | 1.19 (0.54-2.58) |
|  |  | Other | 0.99 (0.64-1.54) | 1.04 (0.65-1.68) | 0.76 (0.33-1.74) |
|  |  | Unknown | 0.68 (0.39-1.19) | 0.8 (0.44-1.44) | 0.59 (0.24-1.43) |
|  |  | Asian | 2.18 (1.11-4.29) | 2.18 (1.11-4.29) | 0.81 (NA-NA) |
| fluoxetine and psycholeptics | N06CA03 | Black/African American | 0.77 (0.6-1) | 0.79 (0.6-1.05) | 0.62 (0.42-0.94) |
|  |  | Hispanic | 0.91 (0.68-1.23) | 0.96 (0.7-1.31) | 0.81 (0.49-1.33) |
|  |  | Other | 0.88 (0.65-1.19) | 0.94 (0.68-1.3) | 0.65 (0.39-1.06) |
|  |  | Unknown | 0.72 (0.51-1.03) | 0.91 (0.63-1.33) | 0.54 (0.32-0.91) |
|  |  | Asian | 1.08 (0.64-1.83) | 1.08 (0.57-1.23) |  |

**Table S4.** Association between race/ethnicity and receiving a prescription of anti-psychotic, anti-depressant and anti-epileptic medications in MSHS among schizophrenia patients. All analyses are adjusted for patient sex, age, comorbid depression and bipolar disorder, and account for the location of the provider. For comparison, we present additional analyses (1) OR, weight + BMI: also adjusted for weight and BMI, and (2) OR, no comorbidity: excluding all individuals with a diagnosis of either depression or bipolar disorder. Multiple testing was accounted for using false discovery rate.

| **medication** | **ATC code** | **Race/ethnicity** | **OR (CI)** | **P-value** | **Q-value** |
| --- | --- | --- | --- | --- | --- |
| carbamazepine | N03AF01 | Black/African American | 0.52 (0.37-0.73) | 0.00 | 0.00 |
|  |  | Hispanic | 0.98 (0.73-1.32) | 0.90 | 0.92 |
|  |  | Other | 0.92 (0.67-1.25) | 0.59 | 0.72 |
|  |  | Unknown | 0.9 (0.67-1.2) | 0.47 | 0.59 |
|  |  | Black/African American | 0.59 (0.44-0.78) | 0.00 | 0.00 |
| oxcarbazepine | N03AF02 | Hispanic | 0.87 (0.66-1.14) | 0.31 | 0.40 |
|  |  | Other | 0.75 (0.55-1) | 0.05 | 0.09 |
|  |  | Unknown | 0.95 (0.73-1.23) | 0.69 | 0.78 |
|  |  | Asian | 0.89 (0.49-1.59) | 0.69 | 0.78 |
| valproic acid | N03AG01 | Black/African American | 1.47 (1.14-1.9) | 0.00 | 0.01 |
|  |  | Hispanic | 1.51 (1.14-2) | 0.00 | 0.01 |
|  |  | Other | 1.32 (0.99-1.76) | 0.06 | 0.10 |
|  |  | Unknown | 0.55 (0.36-0.84) | 0.01 | 0.01 |
|  |  | Asian | 1.2 (0.6-2.38) | 0.61 | 0.72 |
| lamotrigine | N03AX09 | Black/African American | 0.32 (0.28-0.36) | 0.00 | 0.00 |
|  |  | Hispanic | 0.48 (0.43-0.54) | 0.00 | 0.00 |
|  |  | Other | 0.5 (0.45-0.57) | 0.00 | 0.00 |
|  |  | Unknown | 0.75 (0.68-0.83) | 0.00 | 0.00 |
|  |  | Asian | 0.72 (0.57-0.91) | 0.01 | 0.02 |
| chlorpromazine | N05AA01 | Black/African American | 1.76 (1.34-2.32) | 0.00 | 0.00 |
|  |  | Hispanic | 1.91 (1.42-2.58) | 0.00 | 0.00 |
|  |  | Other | 1.41 (1.04-1.91) | 0.03 | 0.05 |
|  |  | Unknown | 0.69 (0.44-1.07) | 0.10 | 0.16 |
|  |  | Asian | 1.2 (0.61-2.35) | 0.59 | 0.72 |
| fluphenazine | N05AB02 | Black/African American | 1.72 (1.2-2.45) | 0.00 | 0.01 |
|  |  | Hispanic | 1.88 (1.28-2.76) | 0.00 | 0.00 |
|  |  | Other | 1.08 (0.7-1.69) | 0.72 | 0.79 |
|  |  | Unknown | 0.97 (0.59-1.6) | 0.91 | 0.92 |
| perphenazine | N05AB03 | Black/African American | 0.76 (0.5-1.14) | 0.19 | 0.28 |
|  |  | Hispanic | 0.59 (0.36-0.97) | 0.04 | 0.07 |
|  |  | Other | 0.89 (0.57-1.39) | 0.60 | 0.72 |
|  |  | Unknown | 0.95 (0.63-1.45) | 0.83 | 0.88 |
| haloperidol | N05AD01 | Black/African American | 1.69 (1.48-1.92) | 0.00 | 0.00 |
|  |  | Hispanic | 1.6 (1.38-1.85) | 0.00 | 0.00 |
|  |  | Other | 1.37 (1.18-1.59) | 0.00 | 0.00 |
|  |  | Unknown | 0.81 (0.66-0.98) | 0.03 | 0.06 |
|  |  | Asian | 1.24 (0.89-1.71) | 0.20 | 0.29 |
| ziprasidone | N05AE04 | Black/African American | 0.92 (0.73-1.15) | 0.45 | 0.57 |
|  |  | Hispanic | 1.12 (0.88-1.41) | 0.36 | 0.46 |
|  |  | Other | 0.85 (0.65-1.11) | 0.22 | 0.32 |
|  |  | Unknown | 0.74 (0.57-0.97) | 0.03 | 0.06 |
|  |  | Asian | 0.87 (0.48-1.55) | 0.63 | 0.73 |
| lurasidone | N05AE05 | Black/African American | 0.62 (0.51-0.76) | 0.00 | 0.00 |
|  |  | Hispanic | 0.77 (0.63-0.93) | 0.01 | 0.02 |
|  |  | Other | 0.65 (0.52-0.82) | 0.00 | 0.00 |
|  |  | Unknown | 0.68 (0.56-0.84) | 0.00 | 0.00 |
|  |  | Asian | 0.76 (0.5-1.15) | 0.19 | 0.28 |
| clozapine | N05AH02 | Black/African American | 0.47 (0.33-0.67) | 0.00 | 0.00 |
|  |  | Hispanic | 0.57 (0.39-0.85) | 0.00 | 0.01 |
|  |  | Other | 0.49 (0.33-0.74) | 0.00 | 0.00 |
|  |  | Unknown | 0.6 (0.4-0.9) | 0.01 | 0.03 |
|  |  | Asian | 1.17 (0.61-2.23) | 0.64 | 0.74 |
| olanzapine | N05AH03 | Black/African American | 1.18 (1.04-1.33) | 0.01 | 0.02 |
|  |  | Hispanic | 1.21 (1.06-1.38) | 0.01 | 0.01 |
|  |  | Other | 1.02 (0.88-1.17) | 0.81 | 0.87 |
|  |  | Unknown | 0.81 (0.69-0.94) | 0.01 | 0.02 |
|  |  | Asian | 1.38 (1.04-1.83) | 0.03 | 0.05 |
| quetiapine | N05AH04 | Black/African American | 1.34 (1.21-1.48) | 0.00 | 0.00 |
|  |  | Hispanic | 1.48 (1.34-1.64) | 0.00 | 0.00 |
|  |  | Other | 1.2 (1.07-1.34) | 0.00 | 0.00 |
|  |  | Unknown | 0.94 (0.84-1.05) | 0.29 | 0.40 |
|  |  | Asian | 1.24 (0.97-1.58) | 0.08 | 0.14 |
| risperidone | N05AX08 | Black/African American | 1.77 (1.58-2) | 0.00 | 0.00 |
|  |  | Hispanic | 1.65 (1.45-1.88) | 0.00 | 0.00 |
|  |  | Other | 1.48 (1.29-1.69) | 0.00 | 0.00 |
|  |  | Unknown | 1.09 (0.94-1.26) | 0.25 | 0.35 |
|  |  | Asian | 1.39 (1.05-1.85) | 0.02 | 0.05 |
| aripiprazole | N05AX12 | Black/African American | 1.15 (1.03-1.28) | 0.01 | 0.03 |
|  |  | Hispanic | 1.23 (1.1-1.38) | 0.00 | 0.00 |
|  |  | Other | 0.98 (0.86-1.11) | 0.70 | 0.78 |
|  |  | Unknown | 0.85 (0.75-0.96) | 0.01 | 0.02 |
|  |  | Asian | 1.14 (0.89-1.47) | 0.30 | 0.40 |
| paliperidone | N05AX13 | Black/African American | 1.48 (1.15-1.92) | 0.00 | 0.01 |
|  |  | Hispanic | 2 (1.54-2.6) | 0.00 | 0.00 |
|  |  | Other | 1.42 (1.07-1.89) | 0.02 | 0.03 |
|  |  | Unknown | 0.87 (0.6-1.26) | 0.45 | 0.57 |
|  |  | Asian | 1.12 (0.62-2.04) | 0.70 | 0.78 |
| cariprazine | N05AX15 | Black/African American | 0.35 (0.16-0.76) | 0.01 | 0.02 |
|  |  | Hispanic | 0.57 (0.29-1.11) | 0.10 | 0.16 |
|  |  | Other | 0.69 (0.35-1.39) | 0.30 | 0.40 |
|  |  | Unknown | 0.9 (0.51-1.57) | 0.71 | 0.78 |
| amitryptiline | N06AA09 | Black/African American | 1.44 (1.09-1.89) | 0.01 | 0.02 |
|  |  | Hispanic | 1.88 (1.44-2.45) | 0.00 | 0.00 |
|  |  | Other | 1.09 (0.78-1.52) | 0.61 | 0.72 |
|  |  | Unknown | 0.71 (0.49-1.02) | 0.06 | 0.11 |
| nortriptyline | N06AA10 | Black/African American | 1.08 (0.73-1.6) | 0.69 | 0.78 |
|  |  | Hispanic | 1.26 (0.86-1.85) | 0.24 | 0.34 |
|  |  | Other | 0.96 (0.61-1.52) | 0.87 | 0.90 |
|  |  | Unknown | 0.58 (0.35-0.98) | 0.04 | 0.07 |
| citalopram | N06AB04 | Black/African American | 1.45 (1.21-1.73) | 0.00 | 0.00 |
|  |  | Hispanic | 1.49 (1.23-1.8) | 0.00 | 0.00 |
|  |  | Other | 1.2 (0.97-1.48) | 0.09 | 0.15 |
|  |  | Unknown | 1.04 (0.83-1.3) | 0.75 | 0.81 |
| paroxetine | N06AB05 | Black/African American | 1.01 (0.77-1.33) | 0.95 | 0.95 |
|  |  | Hispanic | 1.56 (1.21-2.02) | 0.00 | 0.00 |
|  |  | Other | 1.12 (0.82-1.51) | 0.48 | 0.59 |
|  |  | Unknown | 1.02 (0.76-1.38) | 0.88 | 0.90 |
| sertraline | N06AB06 | Black/African American | 1.2 (1.05-1.37) | 0.01 | 0.01 |
|  |  | Hispanic | 1.39 (1.22-1.59) | 0.00 | 0.00 |
|  |  | Other | 1.01 (0.87-1.18) | 0.87 | 0.90 |
|  |  | Unknown | 0.83 (0.71-0.97) | 0.02 | 0.04 |
|  |  | Asian | 0.81 (0.56-1.17) | 0.26 | 0.36 |
| escitalopram | N06AB10 | Black/African American | 0.8 (0.69-0.93) | 0.00 | 0.01 |
|  |  | Hispanic | 1.03 (0.89-1.19) | 0.74 | 0.80 |
|  |  | Other | 0.92 (0.78-1.08) | 0.30 | 0.40 |
|  |  | Unknown | 0.71 (0.6-0.84) | 0.00 | 0.00 |
|  |  | Asian | 1.04 (0.74-1.46) | 0.83 | 0.88 |
| trazodone | N06AX05 | Black/African American | 1.38 (1.2-1.58) | 0.00 | 0.00 |
|  |  | Hispanic | 1.72 (1.5-1.97) | 0.00 | 0.00 |
|  |  | Other | 1.18 (1.01-1.38) | 0.04 | 0.07 |
|  |  | Unknown | 0.8 (0.66-0.96) | 0.02 | 0.03 |
|  |  | Asian | 1.02 (0.71-1.47) | 0.90 | 0.92 |
| mirtazapine | N06AX11 | Black/African American | 1.63 (1.38-1.92) | 0.00 | 0.00 |
|  |  | Hispanic | 2.14 (1.82-2.52) | 0.00 | 0.00 |
|  |  | Other | 1.49 (1.23-1.8) | 0.00 | 0.00 |
|  |  | Unknown | 0.92 (0.74-1.15) | 0.48 | 0.59 |
|  |  | Asian | 0.96 (0.57-1.6) | 0.86 | 0.90 |
| bupropion | N06AX12 | Black/African American | 0.7 (0.62-0.79) | 0.00 | 0.00 |
|  |  | Hispanic | 0.78 (0.69-0.89) | 0.00 | 0.00 |
|  |  | Other | 0.63 (0.55-0.73) | 0.00 | 0.00 |
|  |  | Unknown | 0.8 (0.71-0.91) | 0.00 | 0.00 |
|  |  | Asian | 0.68 (0.5-0.92) | 0.01 | 0.03 |
| venlafaxine | N06AX16 | Black/African American | 0.57 (0.46-0.7) | 0.00 | 0.00 |
|  |  | Hispanic | 0.61 (0.49-0.75) | 0.00 | 0.00 |
|  |  | Other | 0.66 (0.53-0.83) | 0.00 | 0.00 |
|  |  | Unknown | 0.81 (0.66-1) | 0.05 | 0.08 |
|  |  | Asian | 0.65 (0.39-1.07) | 0.09 | 0.15 |
| duloxetine | N06AX21 | Black/African American | 0.81 (0.67-0.97) | 0.02 | 0.04 |
|  |  | Hispanic | 1.12 (0.94-1.34) | 0.19 | 0.28 |
|  |  | Other | 0.72 (0.58-0.9) | 0.00 | 0.01 |
|  |  | Unknown | 0.7 (0.56-0.86) | 0.00 | 0.00 |
|  |  | Asian | 0.68( 0.41-1.14) | 0.15 | 0.22 |
| desvenlafaxine | N06AX23 | Black/African American | 0.44 (0.25-0.78) | 0.01 | 0.01 |
|  |  | Hispanic | 0.41 (0.23-0.76) | 0.00 | 0.01 |
|  |  | Other | 0.48 (0.24-0.94) | 0.03 | 0.06 |
|  |  | Unknown | 0.66 (0.38-1.12) | 0.12 | 0.19 |
| vortioxetine | N06AX26 | Black/African American | 0.13 (0.03-0.48) | 0.00 | 0.01 |
|  |  | Hispanic | 0.67 (0.33-1.32) | 0.25 | 0.34 |
|  |  | Other | 0.51 (0.22-1.21) | 0.13 | 0.19 |
|  |  | Unknown | 0.7 (0.36-1.37) | 0.30 | 0.40 |
| fluoxetine and psycholeptics | N06CA03 | Black/African American | 0.85 (0.73-0.99) | 0.04 | 0.08 |
|  |  | Hispanic | 0.92 (0.78-1.08) | 0.32 | 0.41 |
|  |  | Other | 0.87 (0.73-1.03) | 0.11 | 0.17 |
|  |  | Unknown | 0.71 (0.59-0.86) | 0.00 | 0.00 |
|  |  | Asian | 0.73 (0.49-1.1) | 0.13 | 0.20 |

**Table S5.** Association between race/ethnicity and receiving a prescription of anti-psychotic, anti-depressant and anti-epileptic medications in MSHS among bipolar disorder patients. All analyses are adjusted for patient sex, age, comorbid depression and bipolar disorder, and account for the location of the provider. Multiple testing was accounted for using false discovery rate

| **medication** | **ATC code** | **Race/ethnicity** | **OR (CI)** | **P-value** | **Q-value** |
| --- | --- | --- | --- | --- | --- |
| carbamazepine | N03AF01 | Black/African American | 1.49 (1.19-1.87) | 0.00 | 0.00 |
|  |  | Hispanic | 1.46 (1.18-1.8) | 0.00 | 0.00 |
|  |  | Other | 1.2 (0.94-1.54) | 0.14 | 0.19 |
|  |  | Unknown | 0.94 (0.74-1.19) | 0.61 | 0.67 |
|  |  | Asian | 0.98 (0.56-1.73) | 0.95 | 0.97 |
| oxcarbazepine | N03AF02 | Black/African American | 1.1 (0.9-1.36) | 0.35 | 0.41 |
|  |  | Hispanic | 1.09 (0.9-1.33) | 0.38 | 0.44 |
|  |  | Other | 0.83 (0.66-1.05) | 0.13 | 0.17 |
|  |  | Unknown | 0.76 (0.62-0.93) | 0.01 | 0.01 |
|  |  | Asian | 0.8 (0.49-1.3) | 0.34 | 0.41 |
| valproic acid | N03AG01 | Black/African American | 1.37 (1.08-1.72) | 0.01 | 0.01 |
|  |  | Hispanic | 1.56 (1.25-1.95) | 0.00 | 0.00 |
|  |  | Other | 1.3 (1.07-1.74) | 0.01 | 0.02 |
|  |  | Unknown | 0.53 (0.38-0.76) | 0.00 | 0.00 |
|  |  | Asian | 0.85 (0.46-1.57) | 0.58 | 0.64 |
| lamotrigine | N03AX09 | Black/African American | 0.43 (0.39-0.48) | 0.00 | 0.00 |
|  |  | Hispanic | 0.48 (0.44-0.52) | 0.00 | 0.00 |
|  |  | Other | 0.56 (0.51-0.62) | 0.00 | 0.00 |
|  |  | Unknown | 0.72 (0.67-0.77) | 0.00 | 0.00 |
|  |  | Asian | 0.6 (0.51-0.72) | 0.00 | 0.00 |
| chlorpromazine | N05AA01 | Black/African American | 1.64 (1.3-2.06) | 0.00 | 0.00 |
|  |  | Hispanic | 1.57 (1.24-1.98) | 0.00 | 0.00 |
|  |  | Other | 1.26 (0.98-1.61) | 0.08 | 0.11 |
|  |  | Unknown | 0.64 (0.45-0.89) | 0.01 | 0.02 |
|  |  | Asian | 1.04 (0.59-1.84) | 0.89 | 0.93 |
| fluphenazine | N05AB02 | Black/African American | 1.48 (1.01-2.18) | 0.05 | 0.06 |
|  |  | Hispanic | 1.25 (0.83-1.89) | 0.28 | 0.34 |
|  |  | Other | 1.31 (0.85-2.01) | 0.22 | 0.29 |
|  |  | Unknown | 0.97 (0.58-1.62) | 0.90 | 0.93 |
| perphenazine | N05AB03 | Black/African American | 1.3 (0.92-1.83) | 0.14 | 0.18 |
|  |  | Hispanic | 1.18 (0.84-1.66) | 0.33 | 0.39 |
|  |  | Other | 1.23 (0.85-1.79) | 0.27 | 0.34 |
|  |  | Unknown | 0.78 (0.5-1.21) | 0.27 | 0.34 |
| haloperidol | N05AD01 | Black/African American | 1.89 (1.74-2.06) | 0.00 | 0.00 |
|  |  | Hispanic | 1.59 (1.46-1.74) | 0.00 | 0.00 |
|  |  | Other | 1.42(1.29-1.56) | 0.00 | 0.00 |
|  |  | Unknown | 0.76 (0.67-0.86) | 0.00 | 0.00 |
|  |  | Asian | 1.06 (0.86-1.31) | 0.62 | 0.68 |
| ziprasidone | N05AE04 | Black/African American | 1.25 (1.01-1.56) | 0.04 | 0.06 |
|  |  | Hispanic | 1.13 (0.91-1.4) | 0.27 | 0.34 |
|  |  | Other | 1.01 (0.78-1.3) | 0.96 | 0.97 |
|  |  | Unknown | 0.75 (0.57-0.98) | 0.04 | 0.05 |
|  |  | Asian | 0.71 (0.39-1.3) | 0.27 | 0.34 |
| lurasidone | N05AE05 | Black/African American | 0.7 (0.58-0.86) | 0.00 | 0.00 |
|  |  | Hispanic | 0.83 (0.69-0.99) | 0.04 | 0.06 |
|  |  | Other | 0.66 (0.53-0.83) | 0.00 | 0.00 |
|  |  | Unknown | 0.71 (0.59-0.86) | 0.00 | 0.00 |
|  |  | Asian | 0.72 (0.49-1.07) | 0.11 | 0.15 |
| clozapine | N05AH02 | Black/African American | 0.42 (0.3-0.6) | 0.00 | 0.00 |
|  |  | Hispanic | 0.4 (0.27-0.58) | 0.00 | 0.00 |
|  |  | Other | 0.37 (0.24-0.56) | 0.00 | 0.00 |
|  |  | Unknown | 0.48 (0.32-0.72) | 0.00 | 0.00 |
|  |  | Asian | 0.66 (0.33-1.32) | 0.24 | 0.31 |
| olanzapine | N05AH03 | Black/African American | 1.21 (1.1-1.34) | 0.00 | 0.00 |
|  |  | Hispanic | 1.24 (1.13-1.37) | 0.00 | 0.00 |
|  |  | Other | 1.06 (0.95-1.18) | 0.30 | 0.37 |
|  |  | Unknown | 0.81 (0.71-0.91) | 0.00 | 0.00 |
|  |  | Asian | 1.5 (1.5-1.5) | 0.00 | 0.00 |
| quetiapine | N05AH04 | Black/African American | 1.35 (1.27-1.45) | 0.00 | 0.00 |
|  |  | Hispanic | 1.41 (1.33-1.51) | 0.00 | 0.00 |
|  |  | Other | 1.25 (1.17-1.34) | 0.00 | 0.00 |
|  |  | Unknown | 1.01 (0.94-1.08) | 0.81 | 0.87 |
|  |  | Asian | 1.13 (0.98-1.3) | 0.09 | 0.13 |
| risperidone | N05AX08 | Black/African American | 2 (1.82-2.19) | 0.00 | 0.00 |
|  |  | Hispanic | 1.93 (1.77-2.11) | 0.00 | 0.00 |
|  |  | Other | 1.75 (1.59-1.93) | 0.00 | 0.00 |
|  |  | Unknown | 1.22 (1.1-1.36) | 0.00 | 0.00 |
|  |  | Asian | 1.34 (1.09-1.64) | 0.01 | 0.01 |
| aripiprazole | N05AX12 | Black/African American | 1.08 (1-1.17) | 0.04 | 0.05 |
|  |  | Hispanic | 1.09 (1.01-1.17) | 0.02 | 0.03 |
|  |  | Other | 0.99 (0.91-1.07) | 0.80 | 0.86 |
|  |  | Unknown | 0.85 (0.79-0.92) | 0.00 | 0.00 |
|  |  | Asian | 1 (0.86-1.16) | 0.99 | 0.99 |
| paliperidone | N05AX13 | Black/African American | 1.94 (1.41-2.66) | 0.00 | 0.00 |
|  |  | Hispanic | 1.71 (1.23-2.37) | 0.00 | 0.00 |
|  |  | Other | 1.53 (1.07-2.19) | 0.02 | 0.03 |
|  |  | Unknown | 0.8 (0.49-1.32) | 0.40 | 0.45 |
| cariprazine | N05AX15 | Black/African American | 0.38 (0.19-0.77) | 0.02 | 0.03 |
|  |  | Hispanic | 0.85 (0.52-1.39) | 0.56 | 0.63 |
|  |  | Other | 0.71 (0.39-1.31) | 0.33 | 0.39 |
|  |  | Unknown | 0.74 (0.45-1.21) | 0.29 | 0.35 |
| brexpiprazole | N05AX16 | Black/African American | 0.32 (0.16-0.65) | 0.00 | 0.00 |
|  |  | Hispanic | 0.45 (0.25-0.79) | 0.01 | 0.01 |
|  |  | Other | 0.41 (0.21-0.8) | 0.01 | 0.01 |
|  |  | Unknown | 1.03 (0.71-1.5) | 0.86 | 0.91 |
| desipramine | N06AA01 | Black/African American | 0.4 (0.21-0.77) | 0.01 | 0.01 |
|  |  | Hispanic | 0.51 (0.3-0.84) | 0.01 | 0.01 |
|  |  | Other | 0.55 (0.32-0.97) | 0.04 | 0.06 |
|  |  | Unknown | 0.5 (0.31-0.8) | 0.00 | 0.01 |
| imipramine | N06AA02 | Black/African American | 1 (0.67-1.48) | 0.98 | 0.98 |
|  |  | Hispanic | 1.06 (0.75-1.49) | 0.76 | 0.82 |
|  |  | Other | 0.92 (0.61-1.37) | 0.70 | 0.76 |
|  |  | Unknown | 0.82 (0.58-1.16) | 0.28 | 0.34 |
| clomipramine | N06AA04 | Black/African American | 0.29 (0.16-0.52) | 0.00 | 0.00 |
|  |  | Hispanic | 0.21 (0.12-0.39) | 0.00 | 0.00 |
|  |  | Other | 0.42 (0.25-0.69) | 0.00 | 0.00 |
|  |  | Unknown | 0.56 (0.39-0.82) | 0.00 | 0.01 |
| amitryptiline | N06AA09 | Black/African American | 1.77 (1.61-1.94) | 0.00 | 0.00 |
|  |  | Hispanic | 2.24 (2.06-2.43) | 0.00 | 0.00 |
|  |  | Other | 1.49 (1.35-1.64) | 0.00 | 0.00 |
|  |  | Unknown | 0.81 (0.73-0.89) | 0.00 | 0.00 |
|  |  | Asian | 1.31 (1.07-1.6) | 0.01 | 0.01 |
| nortriptyline | N06AA10 | Black/African American | 1.46 (1.29-1.65) | 0.00 | 0.00 |
|  |  | Hispanic | 1.78 (1.6-1.99) | 0.00 | 0.00 |
|  |  | Other | 1.11 (0.97-1.27) | 0.15 | 0.20 |
|  |  | Unknown | 0.78 (0.69-0.89) | 0.00 | 0.00 |
|  |  | Asian | 1.03 (0.78-1.36) | 0.84 | 0.89 |
| citalopram | N06AB04 | Black/African American | 1.25 (1.17-1.33) | 0.00 | 0.00 |
|  |  | Hispanic | 1.33 (1.26-1.41) | 0.00 | 0.00 |
|  |  | Other | 1.25 (1.17-1.33) | 0.00 | 0.00 |
|  |  | Unknown | 1.02 (0.97-1.08) | 0.45 | 0.51 |
|  |  | Asian | 0.94 (0.82-1.08) | 0.37 | 0.43 |
| paroxetine | N06AB05 | Black/African American | 1 (0.91-1.11) | 0.94 | 0.97 |
|  |  | Hispanic | 1.28 (1.18-1.39) | 0.00 | 0.00 |
|  |  | Other | 1.23 (1.12-1.35) | 0.00 | 0.00 |
|  |  | Unknown | 1.03 (0.94-1.11) | 0.54 | 0.60 |
|  |  | Asian | 1.05 (0.87-1.26) | 0.63 | 0.69 |
| sertraline | N06AB06 | Black/African American | 1.07 (1.02-1.12) | 0.00 | 0.01 |
|  |  | Hispanic | 1.28 (1.23-1.34) | 0.00 | 0.00 |
|  |  | Other | 1.07 (1.02-1.13) | 0.00 | 0.01 |
|  |  | Unknown | 0.93 (0.89-0.97) | 0.00 | 0.00 |
|  |  | Asian | 1.06 (0.97-1.16) | 0.17 | 0.23 |
| fluvoxamine | N06AB08 | Black/African American | 0.24 (0.16-0.38) | 0.00 | 0.00 |
|  |  | Hispanic | 0.38 (0.27-0.52) | 0.00 | 0.00 |
|  |  | Other | 0.38 (0.26-0.54) | 0.00 | 0.00 |
|  |  | Unknown | 0.59 (0.46-0.75) | 0.00 | 0.00 |
|  |  | Asian | 0.81 (0.52-1.26) | 0.33 | 0.40 |
| escitalopram | N06AB10 | Black/African American | 0.78 (0.74-0.82) | 0.00 | 0.00 |
|  |  | Hispanic | 1 (0.96-1.05) | 0.91 | 0.94 |
|  |  | Other | 0.87 (0.83-0.92) | 0.00 | 0.00 |
|  |  | Unknown | 0.84 (0.8-0.87) | 0.00 | 0.00 |
|  |  | Asian | 1 (0.92-1.09) | 0.98 | 0.98 |
| trazodone | N06AX05 | Black/African American | 1.21 (1.13-1.3) | 0.00 | 0.00 |
|  |  | Hispanic | 1.56 (1.46-1.66) | 0.00 | 0.00 |
|  |  | Other | 1.15 (1.07-1.24) | 0.00 | 0.00 |
|  |  | Unknown | 0.77 (0.71-0.83) | 0.00 | 0.00 |
|  |  | Asian | 1.09 (0.95-1.26) | 0.23 | 0.30 |
| mirtazapine | N06AX11 | Black/African American | 1.69 (1.58-1.8) | 0.00 | 0.00 |
|  |  | Hispanic | 1.92 (1.81-2.03) | 0.00 | 0.00 |
|  |  | Other | 1.44 (1.35-1.54) | 0.00 | 0.00 |
|  |  | Unknown | 0.96 (0.9-1.03) | 0.26 | 0.34 |
|  |  | Asian | 1.74 (1.53-1.97) | 0.00 | 0.00 |
| bupropion | N06AX12 | Black/African American | 0.69 (0.66-0.73) | 0.00 | 0.00 |
|  |  | Hispanic | 0.64 (0.61-0.67) | 0.00 | 0.00 |
|  |  | Other | 0.64 (0.61-0.68) | 0.00 | 0.00 |
|  |  | Unknown | 0.78 (0.75-0.81) | 0.00 | 0.00 |
|  |  | Asian | 0.69 (0.63-0.75) | 0.00 | 0.00 |
| venlafaxine | N06AX16 | Black/African American | 0.59 (0.55-0.64) | 0.00 | 0.00 |
|  |  | Hispanic | 0.66 (0.61-0.7) | 0.00 | 0.00 |
|  |  | Other | 0.61 (0.57-0.66) | 0.00 | 0.00 |
|  |  | Unknown | 0.78 (0.73-0.83) | 0.00 | 0.00 |
|  |  | Asian | 0.81 (0.71-0.93) | 0.00 | 0.00 |
| duloxetine | N06AX21 | Black/African American | 1.11 (1.04-1.18) | 0.00 | 0.00 |
|  |  | Hispanic | 1.24 (1.17-1.31) | 0.00 | 0.00 |
|  |  | Other | 0.92 (0.86-0.99) | 0.02 | 0.04 |
|  |  | Unknown | 0.76 (0.72-0.81) | 0.00 | 0.00 |
|  |  | Asian | 0.75 (0.65-0.86) | 0.00 | 0.00 |
| desvenlafaxine | N06AX23 | Black/African American | 0.33 (0.26-0.42) | 0.00 | 0.00 |
|  |  | Hispanic | 0.38 (0.31-0.47) | 0.00 | 0.00 |
|  |  | Other | 0.5 (0.41-0.62) | 0.00 | 0.00 |
|  |  | Unknown | 0.71 (0.61-0.81) | 0.00 | 0.00 |
|  |  | Asian | 0.59 (0.41-0.84) | 0.00 | 0.01 |
| vilazodone | N06AX24 | Black/African American | 0.2 (0.13-0.33) | 0.00 | 0.00 |
|  |  | Hispanic | 0.24 (0.16-0.35) | 0.00 | 0.00 |
|  |  | Other | 0.42 (0.3-0.59) | 0.00 | 0.00 |
|  |  | Unknown | 0.71 (0.58-0.88) | 0.00 | 0.00 |
|  |  | Asian | 0.56 (0.32-0.98) | 0.04 | 0.06 |
| vortioxetine | N06AX26 | Black/African American | 0.21 (0.15-0.31) | 0.00 | 0.00 |
|  |  | Hispanic | 0.54 (0.44-0.67) | 0.00 | 0.00 |
|  |  | Other | 0.43 (0.33-0.57) | 0.00 | 0.00 |
|  |  | Unknown | 0.69 (0.59-0.82) | 0.00 | 0.00 |
|  |  | Asian | 0.67 (0.45-0.99) | 0.04 | 0.05 |
| fluoxetine and psycholeptics | N06CA03 | Black/African American | 0.74 (0.7-0.79) | 0.00 | 0.00 |
|  |  | Hispanic | 0.89 (0.84-0.94) | 0.00 | 0.00 |
|  |  | Other | 0.85 (0.8-0.9) | 0.00 | 0.00 |
|  |  | Unknown | 0.85 (0.81-0.9) | 0.00 | 0.00 |
|  |  | Asian | 0.82 (0.74-0.92) | 0.00 | 0.00 |

**Table S6.** Association between race/ethnicity and receiving a prescription of anti-psychotic, anti-depressant and anti-epileptic medications in MSHS among depression patients. All analyses are adjusted for patient sex, age, comorbid depression and bipolar disorder, and account for the location of the provider. Multiple testing was accounted for using false discovery rate.

| **medication** | **ATC code** | **Race /**  **ethnicity** | **Beta,**  **Main** | **Beta,**  **weight + BMI** | **Beta**  **no comorbidity** |
| --- | --- | --- | --- | --- | --- |
| oxcarbazepine | N03AF02 | Asian | 0.02 | NA | NA |
|  |  | Black/African American | 0.24 | 0.3 | NA |
|  |  | Hispanic | 0.20 | -0.01 | NA |
|  |  | Other | 0.30 | 0.42 | NA |
|  |  | Unknown | 0.13 | 0.1 | NA |
| valproic acid | N03AG01 | Asian | 0.09 | 0.12 | 0.02 |
|  |  | Black/African American | -0.15 | -0.25 | -0.34 |
|  |  | Hispanic | -0.29 | -0.39 | -0.22 |
|  |  | Other | -0.23 | -0.37 | -0.25 |
|  |  | Unknown | -0.03 | -0.02 | -0.13 |
| lamotrigine | N03AX09 | Asian | 0.41 | 0.36 | NA |
|  |  | Black/African American | -0.14 | -0.2 | -0.22 |
|  |  | Hispanic | -0.27 | -0.26 | -0.16 |
|  |  | Other | -0.50 | -0.57 | -0.25 |
|  |  | Unknown | 0.12 | 0.03 | 0.38 |
| fluphenazine | N05AB02 | Asian | 0.13 | 0.18 | 0.34 |
|  |  | Black/African American | 0.14 | 0.13 | 0.13 |
|  |  | Hispanic | 0.11 | 0.1 | 0.2 |
|  |  | Other | 0.24 | 0.2 | 0.23 |
|  |  | Unknown | -0.18 | -0.22 | -0.15 |
| haloperidol | N05AD01 | Asian | -0.07 | -0.05 | -0.01 |
|  |  | Black/African American | -0.08 | -0.08 | -0.1 |
|  |  | Hispanic | -0.04 | -0.01 | -0.05 |
|  |  | Other | -0.07 | -0.07 | -0.06 |
|  |  | Unknown | -0.15 | -0.14 | -0.16 |
| ziprasidone | N05AE04 | Asian | -0.06 | -0.01 | NA |
|  |  | Black/African American | -0.14 | -0.22 | -0.12 |
|  |  | Hispanic | -0.23 | -0.28 | -0.4 |
|  |  | Other | -0.11 | -0.1 | -0.17 |
|  |  | Unknown | -0.49 | -0.53 | -0.4 |
| clozapine | N05AH02 | Asian | -0.27 | -0.22 | -0.35 |
|  |  | Black/African American | 0.04 | 0.09 | 0 |
|  |  | Hispanic | -0.02 | 0.02 | -0.07 |
|  |  | Other | -0.13 | -0.06 | -0.21 |
|  |  | Unknown | -0.22 | -0.17 | -0.28 |
| olanzapine | N05AH03 | Asian | -0.03 | 0.14 | -0.07 |
|  |  | Black/African American | 0.05 | 0.1 | -0.03 |
|  |  | Hispanic | 0.08 | 0.15 | 0.06 |
|  |  | Other | 0.03 | 0.08 | 0 |
|  |  | Unknown | -0.02 | 0 | -0.03 |
| quetiapine | N05AH04 | Asian | -0.11 | -0.05 | -0.14 |
|  |  | Black/African American | 0.14 | 0.13 | 0.05 |
|  |  | Hispanic | 0.06 | 0.08 | 0.15 |
|  |  | Other | 0.09 | 0.14 | 0.11 |
|  |  | Unknown | 0.06 | 0.15 | 0.04 |
| risperidone | N05AX08 | Asian | -0.01 | 0.03 | 0.06 |
|  |  | Black/African American | -0.02 | -0.04 | 0.03 |
|  |  | Hispanic | -0.04 | -0.05 | -0.01 |
|  |  | Other | 0.03 | 0.02 | 0.06 |
|  |  | Unknown | 0.04 | 0.04 | 0 |
| aripiprazole | N05AX12 | Asian | -0.07 | -0.01 | 0.14 |
|  |  | Black/African American | -0.02 | -0.03 | 0.03 |
|  |  | Hispanic | 0.05 | 0.05 | 0.16 |
|  |  | Other | -0.02 | -0.04 | 0.01 |
|  |  | Unknown | -0.05 | -0.04 | 0.11 |
| paliperidone | N05AX13 | Asian | 0.15 | 0.14 | 0.41 |
|  |  | Black/African American | 0.23 | 0.23 | 0.23 |
|  |  | Hispanic | 0.18 | 0.16 | 0.05 |
|  |  | Other | 0.00 | -0.04 | -0.02 |
|  |  | Unknown | 0.12 | 0.15 | 0.14 |
| sertraline | N06AB06 | Asian | -0.13 | -0.06 | -0.09 |
|  |  | Black/African American | -0.07 | -0.04 | -0.15 |
|  |  | Hispanic | -0.04 | 0.01 | -0.19 |
|  |  | Other | -0.16 | -0.08 | -0.10 |
|  |  | Unknown | -0.05 | -0.09 | -0.15 |
| escitalopram | N06AB10 | Asian | -0.24 | -0.13 | 0.00 |
|  |  | Black/African American | -0.10 | -0.07 | -0.19 |
|  |  | Hispanic | -0.12 | -0.03 | -0.11 |
|  |  | Other | -0.01 | 0.06 | -0.21 |
|  |  | Unknown | -0.09 | 0.07 | -0.44 |
| trazodone | N06AX05 | Asian | -0.28 | -0.28 | -0.13 |
|  |  | Black/African American | -0.07 | -0.07 | -0.16 |
|  |  | Hispanic | -0.09 | -0.12 | -0.16 |
|  |  | Other | -0.03 | -0.05 | -0.04 |
|  |  | Unknown | -0.13 | -0.2 | -0.18 |
| mirtazapine | N06AX11 | Asian | -0.01 | NA | NA |
|  |  | Black/African American | -0.05 | -0.07 | 0.21 |
|  |  | Hispanic | -0.07 | -0.1 | -0.07 |
|  |  | Other | 0.01 | 0 | 0.35 |
|  |  | Unknown | 0.15 | 0.1 | 0.36 |
| bupropion | N06AX12 | Asian | -0.98 | -1.01 | 0.14 |
|  |  | Black/African American | -0.14 | -0.27 | -0.11 |
|  |  | Hispanic | -0.14 | -0.23 | -0.06 |
|  |  | Other | -0.20 | -0.22 | 0.07 |
|  |  | Unknown | -0.22 | -0.2 | -0.3 |
| duloxetine | N06AX21 | Asian | -0.24 | -0.23 | -0.34 |
|  |  | Black/African American | -0.10 | 0.02 | -0.61 |
|  |  | Hispanic | -0.02 | 0.02 | 0.27 |
|  |  | Other | 0.33 | 0.43 | 0.03 |
|  |  | Unknown | -0.40 | -0.41 | -0.74 |
| fluoxetine and psycholeptics | N06CA03 | Asian | -0.13 | 0.12 | NA |
|  |  | Black/African American | -0.22 | -0.16 | -0.34 |
|  |  | Hispanic | -0.20 | -0.2 | -0.03 |
|  |  | Other | -0.21 | -0.25 | -0.04 |
|  |  | Unknown | 0.05 | 0.07 | 0.07 |

**Table S7.** Association between race/ethnicity and the prescribed daily dose of anti-psychotic, anti-depressant and anti-epileptic medications in MSHS among schizophrenia patients. All analyses are adjusted for patient sex, age, comorbid depression and bipolar disorder, and account for the location of the provider. For comparison with the main analyses, we present additional analyses (1) Beta, weight + BMI: also adjusted for weight and BMI, and (2) Beta, no comorbidity: excluding all individuals with a diagnosis of either depression or bipolar disorder. Multiple testing was accounted for using false discovery rate. NE indicate effect sizes in sub-analyses that could not be computed due to too small sample size of patients of specific race/ethnicity taking specific medication.

| **medication** | **ATC code** | **Race/ethnicity** | **Beta** | **P-value** | **Q-value** |
| --- | --- | --- | --- | --- | --- |
| oxcarbazepine | N03AF02 | Asian | -0.40 | 0.135 | 0.393 |
|  |  | Black/African American | 0.07 | 0.605 | 0.784 |
|  |  | Hispanic | 0.27 | 0.020 | 0.106 |
|  |  | Other | -0.08 | 0.551 | 0.758 |
|  |  | Unknown | 0.12 | 0.310 | 0.591 |
| valproic acid | N03AG01 | Asian | 0.27 | 0.419 | 0.712 |
|  |  | Black/African American | 0.24 | 0.054 | 0.216 |
|  |  | Hispanic | 0.04 | 0.785 | 0.887 |
|  |  | Other | 0.11 | 0.406 | 0.712 |
|  |  | Unknown | 0.12 | 0.544 | 0.758 |
| lamotrigine | N03AX09 | Asian | -0.23 | 0.009 | 0.065 |
|  |  | Black/African American | **-0.29** | **0.000** | **0.000** |
|  |  | Hispanic | **-0.24** | **0.000** | **0.000** |
|  |  | Other | **-0.19** | **0.000** | **0.002** |
|  |  | Unknown | -0.02 | 0.646 | 0.798 |
| chlorpromazine | N05AA01 | Asian | 0.16 | 0.646 | 0.798 |
|  |  | Black/African American | 0.10 | 0.427 | 0.712 |
|  |  | Hispanic | 0.02 | 0.876 | 0.929 |
|  |  | Other | 0.03 | 0.853 | 0.929 |
|  |  | Unknown | 0.13 | 0.539 | 0.758 |
| haloperidol | N05AD01 | Asian | 0.05 | 0.405 | 0.712 |
|  |  | Black/African American | 0.03 | 0.232 | 0.508 |
|  |  | Hispanic | 0.00 | 0.887 | 0.931 |
|  |  | Other | -0.01 | 0.754 | 0.864 |
|  |  | Unknown | -0.03 | 0.459 | 0.730 |
| ziprasidone | N05AE04 | Asian | 0.73 | 0.006 | 0.061 |
|  |  | Black/African American | 0.14 | 0.188 | 0.468 |
|  |  | Hispanic | -0.05 | 0.628 | 0.798 |
|  |  | Other | 0.03 | 0.823 | 0.910 |
|  |  | Unknown | 0.01 | 0.961 | 0.989 |
| lurasidone | N05AE05 | Asian | -0.24 | 0.230 | 0.508 |
|  |  | Black/African American | -0.13 | 0.139 | 0.394 |
|  |  | Hispanic | 0.05 | 0.547 | 0.758 |
|  |  | Other | -0.15 | 0.143 | 0.394 |
|  |  | Unknown | 0.07 | 0.439 | 0.720 |
| clozapine | N05AH02 | Asian | -0.06 | 0.797 | 0.890 |
|  |  | Black/African American | 0.10 | 0.447 | 0.722 |
|  |  | Hispanic | 0.25 | 0.074 | 0.263 |
|  |  | Other | 0.31 | 0.048 | 0.205 |
|  |  | Unknown | 0.06 | 0.720 | 0.840 |
| olanzapine | N05AH03 | Asian | -0.05 | 0.653 | 0.798 |
|  |  | Black/African American | **0.17** | **0.000** | **0.004** |
|  |  | Hispanic | 0.09 | 0.082 | 0.278 |
|  |  | Other | 0.08 | 0.146 | 0.394 |
|  |  | Unknown | -0.13 | 0.042 | 0.193 |
| quetiapine | N05AH04 | Asian | -0.06 | 0.563 | 0.758 |
|  |  | Black/African American | **0.14** | **0.001** | **0.015** |
|  |  | Hispanic | 0.12 | 0.005 | 0.051 |
|  |  | Other | 0.08 | 0.096 | 0.305 |
|  |  | Unknown | 0.06 | 0.208 | 0.484 |
| risperidone | N05AX08 | Asian | 0.06 | 0.333 | 0.624 |
|  |  | Black/African American | 0.07 | 0.014 | 0.090 |
|  |  | Hispanic | **0.09** | **0.002** | **0.026** |
|  |  | Other | 0.05 | 0.075 | 0.263 |
|  |  | Unknown | 0.08 | 0.021 | 0.106 |
| aripiprazole | N05AX12 | Asian | -0.06 | 0.376 | 0.692 |
|  |  | Black/African American | 0.02 | 0.467 | 0.731 |
|  |  | Hispanic | 0.02 | 0.508 | 0.758 |
|  |  | Other | 0.03 | 0.305 | 0.591 |
|  |  | Unknown | 0.02 | 0.562 | 0.758 |
| paliperidone | N05AX13 | Asian | -0.03 | 0.876 | 0.929 |
|  |  | Black/African American | 0.16 | 0.108 | 0.334 |
|  |  | Hispanic | 0.12 | 0.216 | 0.493 |
|  |  | Other | 0.07 | 0.500 | 0.758 |
|  |  | Unknown | -0.28 | 0.068 | 0.263 |
| sertraline | N06AB06 | Asian | -0.07 | 0.678 | 0.800 |
|  |  | Black/African American | -0.16 | 0.008 | 0.065 |
|  |  | Hispanic | -0.07 | 0.275 | 0.567 |
|  |  | Other | -0.12 | 0.089 | 0.292 |
|  |  | Unknown | -0.17 | 0.025 | 0.118 |
| escitalopram | N06AB10 | Asian | -0.30 | 0.075 | 0.263 |
|  |  | Black/African American | -0.06 | 0.427 | 0.712 |
|  |  | Hispanic | -0.10 | 0.161 | 0.424 |
|  |  | Other | -0.10 | 0.191 | 0.468 |
|  |  | Unknown | 0.21 | 0.015 | 0.090 |
| trazodone | N06AX05 | Asian | 0.12 | 0.537 | 0.758 |
|  |  | Black/African American | 0.01 | 0.859 | 0.929 |
|  |  | Hispanic | 0.07 | 0.255 | 0.536 |
|  |  | Other | 0.03 | 0.652 | 0.798 |
|  |  | Unknown | 0.04 | 0.664 | 0.800 |
| mirtazapine | N06AX11 | Asian | 0.02 | 0.944 | 0.981 |
|  |  | Black/African American | -0.03 | 0.678 | 0.800 |
|  |  | Hispanic | 0.00 | 0.975 | 0.994 |
|  |  | Other | 0.21 | 0.021 | 0.106 |
|  |  | Unknown | 0.00 | 0.998 | 0.998 |
| bupropion | N06AX12 | Asian | -0.33 | 0.013 | 0.089 |
|  |  | Black/African American | **-0.20** | **0.000** | **0.005** |
|  |  | Hispanic | -0.03 | 0.560 | 0.758 |
|  |  | Other | -0.07 | 0.285 | 0.576 |
|  |  | Unknown | -0.06 | 0.306 | 0.591 |
| venlafaxine | N06AX16 | Asian | -0.62 | 0.008 | 0.065 |
|  |  | Black/African American | -0.18 | 0.049 | 0.205 |
|  |  | Hispanic | -0.05 | 0.589 | 0.783 |
|  |  | Other | -0.13 | 0.197 | 0.469 |
|  |  | Unknown | -0.03 | 0.757 | 0.864 |
| duloxetine | N06AX21 | Asian | 0.13 | 0.599 | 0.784 |
|  |  | Black/African American | **-0.26** | **0.003** | **0.037** |
|  |  | Hispanic | -0.05 | 0.529 | 0.758 |
|  |  | Other | -0.12 | 0.240 | 0.515 |
|  |  | Unknown | -0.08 | 0.416 | 0.712 |
| fluoxetine and psycholeptics | N06CA03 | Asian | 0.00 | 0.995 | 0.998 |
|  |  | Black/African American | **-0.19** | **0.004** | **0.045** |
|  |  | Hispanic | -0.16 | 0.018 | 0.104 |
|  |  | Other | -0.10 | 0.192 | 0.468 |
|  |  | Unknown | -0.13 | 0.123 | 0.368 |

**Table S8.** Association between race/ethnicity and the prescribed daily dose of anti-psychotic, anti-depressant and anti-epileptic medications in MSHS among bipolar disorder patients. All analyses are adjusted for patient sex, age, comorbid depression and schizophrenia, and account for the location of the provider. Multiple testing was accounted for using false discovery rate.

| **medication** | **ATC code** | **Race/ethnicity** | **Beta** | **P-value** | **Q-value** |
| --- | --- | --- | --- | --- | --- |
| carbamazepine | N03AF01 | Asian | 0.01 | 0.978 | 0.978 |
|  |  | Black/African American | -0.09 | 0.408 | 0.629 |
|  |  | Hispanic | -0.01 | 0.899 | 0.952 |
|  |  | Other | -0.04 | 0.715 | 0.823 |
|  |  | Unknown | 0.11 | 0.311 | 0.513 |
| oxcarbazepine | N03AF02 | Asian | -0.26 | 0.245 | 0.438 |
|  |  | Black/African American | 0.15 | 0.107 | 0.245 |
|  |  | Hispanic | 0.05 | 0.551 | 0.714 |
|  |  | Other | -0.08 | 0.480 | 0.681 |
|  |  | Unknown | -0.07 | 0.456 | 0.666 |
| valproic acid | N03AG01 | Asian | 0.14 | 0.699 | 0.811 |
|  |  | Black/African American | 0.05 | 0.754 | 0.841 |
|  |  | Hispanic | -0.12 | 0.416 | 0.634 |
|  |  | Other | -0.09 | 0.489 | 0.681 |
|  |  | Unknown | 0.12 | 0.527 | 0.701 |
| lamotrigine | N03AX09 | Asian | -0.05 | 0.526 | 0.701 |
|  |  | Black/African American | **-0.13** | **0.002** | **0.010** |
|  |  | Hispanic | -0.07 | 0.066 | 0.183 |
|  |  | Other | -0.08 | 0.071 | 0.186 |
|  |  | Unknown | 0.00 | 0.888 | 0.952 |
| chlorpromazine | N05AA01 | Asian | -0.01 | 0.971 | 0.978 |
|  |  | Black/African American | 0.10 | 0.224 | 0.417 |
|  |  | Hispanic | -0.05 | 0.594 | 0.727 |
|  |  | Other | 0.04 | 0.669 | 0.789 |
|  |  | Unknown | 0.18 | 0.168 | 0.358 |
| haloperidol | N05AD01 | Asian | -0.04 | 0.191 | 0.382 |
|  |  | Black/African American | 0.02 | 0.192 | 0.382 |
|  |  | Hispanic | 0.01 | 0.589 | 0.727 |
|  |  | Other | -0.01 | 0.434 | 0.647 |
|  |  | Unknown | -0.03 | 0.235 | 0.426 |
| ziprasidone | N05AE04 | Asian | 0.13 | 0.647 | 0.769 |
|  |  | Black/African American | 0.07 | 0.499 | 0.682 |
|  |  | Hispanic | 0.13 | 0.192 | 0.382 |
|  |  | Other | 0.19 | 0.102 | 0.245 |
|  |  | Unknown | 0.22 | 0.068 | 0.183 |
| lurasidone | N05AE05 | Asian | -0.02 | 0.913 | 0.959 |
|  |  | Black/African American | 0.01 | 0.935 | 0.962 |
|  |  | Hispanic | 0.01 | 0.921 | 0.961 |
|  |  | Other | 0.02 | 0.864 | 0.935 |
|  |  | Unknown | 0.12 | 0.183 | 0.380 |
| clozapine | N05AH02 | Asian | -0.10 | 0.641 | 0.768 |
|  |  | Black/African American | 0.26 | 0.028 | 0.090 |
|  |  | Hispanic | -0.20 | 0.105 | 0.245 |
|  |  | Other | 0.03 | 0.847 | 0.930 |
|  |  | Unknown | 0.00 | 0.976 | 0.978 |
| olanzapine | N05AH03 | Asian | -0.06 | 0.365 | 0.569 |
|  |  | Black/African American | **0.16** | **0.000** | **0.000** |
|  |  | Hispanic | 0.08 | 0.026 | 0.085 |
|  |  | Other | 0.09 | 0.021 | 0.073 |
|  |  | Unknown | -0.01 | 0.895 | 0.952 |
| quetiapine | N05AH04 | Asian | -0.03 | 0.593 | 0.727 |
|  |  | Black/African American | **0.09** | **0.000** | **0.001** |
|  |  | Hispanic | 0.03 | 0.201 | 0.387 |
|  |  | Other | 0.04 | 0.139 | 0.305 |
|  |  | Unknown | **0.07** | **0.009** | **0.034** |
| risperidone | N05AX08 | Asian | 0.00 | 0.932 | 0.962 |
|  |  | Black/African American | 0.02 | 0.224 | 0.417 |
|  |  | Hispanic | 0.03 | 0.048 | 0.139 |
|  |  | Other | 0.02 | 0.203 | 0.387 |
|  |  | Unknown | 0.05 | 0.015 | 0.058 |
| aripiprazole | N05AX12 | Asian | -0.02 | 0.518 | 0.701 |
|  |  | Black/African American | 0.02 | 0.127 | 0.288 |
|  |  | Hispanic | 0.02 | 0.072 | 0.186 |
|  |  | Other | 0.02 | 0.202 | 0.387 |
|  |  | Unknown | -0.01 | 0.685 | 0.801 |
| amitryptiline | N06AA09 | Asian | -0.10 | 0.264 | 0.464 |
|  |  | Black/African American | 0.05 | 0.269 | 0.464 |
|  |  | Hispanic | 0.04 | 0.288 | 0.485 |
|  |  | Other | 0.02 | 0.618 | 0.747 |
|  |  | Unknown | 0.11 | 0.018 | 0.064 |
| nortriptyline | N06AA10 | Asian | 0.12 | 0.359 | 0.566 |
|  |  | Black/African American | -0.08 | 0.179 | 0.377 |
|  |  | Hispanic | **-0.21** | **0.000** | **0.000** |
|  |  | Other | **-0.17** | **0.010** | **0.037** |
|  |  | Unknown | -0.02 | 0.728 | 0.824 |
| citalopram | N06AB04 | Asian | **-0.24** | **0.000** | **0.001** |
|  |  | Black/African American | **-0.15** | **0.000** | **0.000** |
|  |  | Hispanic | **-0.13** | **0.000** | **0.000** |
|  |  | Other | **-0.15** | **0.000** | **0.000** |
|  |  | Unknown | **-0.10** | **0.000** | **0.001** |
| paroxetine | N06AB05 | Asian | **-0.36** | **0.000** | **0.000** |
|  |  | Black/African American | -0.04 | 0.420 | 0.634 |
|  |  | Hispanic | -0.03 | 0.493 | 0.681 |
|  |  | Other | -0.08 | 0.067 | 0.183 |
|  |  | Unknown | -0.11 | 0.007 | 0.028 |
| sertraline | N06AB06 | Asian | **-0.20** | **0.000** | **0.000** |
|  |  | Black/African American | **-0.13** | **0.000** | **0.000** |
|  |  | Hispanic | **-0.13** | **0.000** | **0.000** |
|  |  | Other | **-0.13** | **0.000** | **0.000** |
|  |  | Unknown | **-0.08** | **0.000** | **0.000** |
| fluvoxamine | N06AB08 | Asian | -0.07 | 0.754 | 0.841 |
|  |  | Black/African American | -0.11 | 0.597 | 0.727 |
|  |  | Hispanic | -0.28 | 0.049 | 0.140 |
|  |  | Other | -0.17 | 0.301 | 0.502 |
|  |  | Unknown | -0.08 | 0.459 | 0.666 |
| escitalopram | N06AB10 | Asian | **-0.24** | **0.000** | **0.000** |
|  |  | Black/African American | **-0.07** | **0.001** | **0.004** |
|  |  | Hispanic | **-0.13** | **0.000** | **0.000** |
|  |  | Other | **-0.11** | **0.000** | **0.000** |
|  |  | Unknown | **-0.06** | **0.002** | **0.008** |
| trazodone | N06AX05 | Asian | -0.08 | 0.232 | 0.425 |
|  |  | Black/African American | -0.02 | 0.437 | 0.647 |
|  |  | Hispanic | -0.02 | 0.489 | 0.681 |
|  |  | Other | -0.02 | 0.581 | 0.727 |
|  |  | Unknown | -0.02 | 0.578 | 0.727 |
| mirtazapine | N06AX11 | Asian | **-0.17** | **0.003** | **0.014** |
|  |  | Black/African American | **-0.09** | **0.002** | **0.008** |
|  |  | Hispanic | **-0.11** | **0.000** | **0.000** |
|  |  | Other | -0.06 | 0.035 | 0.106 |
|  |  | Unknown | -0.02 | 0.566 | 0.726 |
| bupropion | N06AX12 | Asian | -0.08 | 0.036 | 0.106 |
|  |  | Black/African American | **-0.12** | **0.000** | **0.000** |
|  |  | Hispanic | **-0.07** | **0.000** | **0.001** |
|  |  | Other | **-0.14** | **0.000** | **0.000** |
|  |  | Unknown | **-0.05** | **0.005** | **0.022** |
| venlafaxine | N06AX16 | Asian | -0.15 | 0.015 | 0.058 |
|  |  | Black/African American | **-0.18** | **0.000** | **0.000** |
|  |  | Hispanic | **-0.19** | **0.000** | **0.000** |
|  |  | Other | -0.06 | 0.131 | 0.292 |
|  |  | Unknown | 0.05 | 0.091 | 0.225 |
| duloxetine | N06AX21 | Asian | -0.06 | 0.353 | 0.563 |
|  |  | Black/African American | -0.05 | 0.082 | 0.210 |
|  |  | Hispanic | **-0.09** | **0.001** | **0.004** |
|  |  | Other | **-0.09** | **0.005** | **0.022** |
|  |  | Unknown | -0.06 | 0.032 | 0.097 |
| desvenlafaxine | N06AX23 | Asian | -0.06 | 0.722 | 0.824 |
|  |  | Black/African American | -0.27 | 0.016 | 0.059 |
|  |  | Hispanic | -0.11 | 0.266 | 0.464 |
|  |  | Other | -0.11 | 0.285 | 0.485 |
|  |  | Unknown | -0.04 | 0.541 | 0.713 |
| vilazodone | N06AX24 | Asian | 0.19 | 0.487 | 0.681 |
|  |  | Black/African American | 0.04 | 0.862 | 0.935 |
|  |  | Hispanic | -0.31 | 0.103 | 0.245 |
|  |  | Other | 0.37 | 0.029 | 0.091 |
|  |  | Unknown | -0.01 | 0.958 | 0.978 |
| vortioxetine | N06AX26 | Asian | -0.26 | 0.166 | 0.358 |
|  |  | Black/African American | -0.11 | 0.547 | 0.714 |
|  |  | Hispanic | -0.10 | 0.334 | 0.544 |
|  |  | Other | 0.13 | 0.338 | 0.545 |
|  |  | Unknown | -0.02 | 0.811 | 0.898 |
| fluoxetine and psycholeptics | N06CA03 | Asian | -0.08 | 0.091 | 0.225 |
|  |  | Black/African American | **-0.08** | **0.005** | **0.022** |
|  |  | Hispanic | -0.05 | 0.024 | 0.080 |
|  |  | Other | **-0.08** | **0.002** | **0.009** |
|  |  | Unknown | **-0.06** | **0.004** | **0.020** |

**Table S9.** Association between race/ethnicity and the prescribed daily dose of anti-psychotic, anti-depressant and anti-epileptic medications in MSHS among depression patients. All analyses are adjusted for patient sex, age, comorbid bipolar and schizophrenia, and account for the location of the provider. Multiple testing was accounted for using false discovery rate.
